# Publisher Correction: Structural basis for the inhibition of HTLV-1 integration inferred from cryo-EM deltaretroviral intasome structures

**DOI:** 10.1038/s41467-021-26243-6

**Published:** 2021-10-05

**Authors:** Michal S. Barski, Teresa Vanzo, Xue Zhi Zhao, Steven J. Smith, Allison Ballandras-Colas, Nora B. Cronin, Valerie E. Pye, Stephen H. Hughes, Terrence R. Burke, Peter Cherepanov, Goedele N. Maertens

**Affiliations:** 1 Imperial College London, St. Mary’s Hospital, Department of Infectious Disease, Section of Virology, Norfolk Place, London, UK; 2 Chemical Biology Laboratory, Centre for Cancer Research, National Cancer Institute, Frederick, MD USA; 3 Retroviral Replication Laboratory, Centre for Cancer Research, National Cancer Institute, Frederick, MD USA; 4 Chromatin Structure & Mobile DNA Laboratory, The Francis Crick Institute, London, UK; 5 LonCEM Facility, The Francis Crick Institute, London, UK; 6 Present Address: International Institute of Molecular Mechanisms and Machines, Polish Academy of Sciences, Warsaw, Poland; 7 Present Address: Department CIBIO, University of Trento, Povo-Trento, Italy

**Keywords:** Retrovirus, Cryoelectron microscopy

Correction to: *Nature Communications* 10.1038/s41467-021-25284-1, published online 17 August 2021.

The original version of this Article contained an error in the author affiliations.

Stephen H. Hughes was incorrectly associated with ‘Chromatin Structure & Mobile DNA Laboratory, The Francis Crick Institute, London, UK’.

This has now been corrected in both the PDF and HTML versions of the Article.

