# Supplementary material for: Structural basis for the inhibition of HTLV-1 integration inferred from cryo-EM deltaretroviral intasome structures
Source: Nat Commun. 2021 Aug 17;12:4996. doi: 10.1038/s41467-021-25284-1 (PMC8370991; doi:10.1038/s41467-021-25284-1)
Supplement: Supplementary file 1 — Supplementary Information [file 41467_2021_25284_MOESM1_ESM.pdf]

Supplementary Information

Structural basis for the inhibition of HTLV-1 integration inferred from cryo-EM deltaretroviral intasome structures

Michal S. Barski[a,1], Teresa Vanzo[a,b,1], Xue Zhi Zhao[c], Steven Smith[d], Allison Ballandras-Colas[e], Nora Cronin[f], Valerie E. Pye[e], Stephen H. Hughes[d], Terrence R. Burke[c], Peter Cherepanov[a,e], and Goedele N. Maertens[a,*]

Supplementary Materials and Methods
Supplementary Figures: 18
Supplementary Tables: 6

Supplementary Materials and Methods

Preparation of Compounds XZ242, XZ256, and XZ420.

General Synthetic Procedures. Proton (^{1}H) and carbon (^{13}C) NMR spectra were recorded on a Varian 400 MHz spectrometer or a Varian 500 MHz spectrometer and are reported in ppm relative to TMS and referenced to the solvent in which the spectra were collected. Wherever mentioned, room temperature (rt) was around 22ºC. Solvent was removed by rotary evaporation under reduced pressure, and anhydrous solvents were obtained commercially and used without further drying. Purification by silica gel chromatography was performed with EtOAc−hexanes solvent systems. Preparative high pressure liquid chromatography (HPLC) was conducted using a Waters Prep LC4000 system having photodiode array detection and Phenomenex C18 columns (catalogue no. 00G4436-P0-AX, 250 mm × 21.2 mm 10 μm particle size, 110 Å pore) at a flow rate of 10 mL/min. Binary solvent systems consisting of A = 0.1% aqueous TFA and B = 0.1% TFA in acetonitrile were employed with gradients as indicated. Products were obtained as amorphous solids following lyophilization. Electrospray ionization-mass spectrometric (ESI-MS) were acquired with an Agilent LC/MSD system equipped with a multimode ion source. High resolution mass spectrometric (HRMS) were acquired by LC/MS-ESI using LTQ-Orbitrap-XL at 30K resolution.

I. Preparation of 5-(3-Chloro-4-fluorobenzyl)-7-hydroxy-2,4-dimethyl-1*H*-pyrrolo[3,4-*c*]pyridine-1,3,6(2*H*,5*H*)-trione (XZ242).

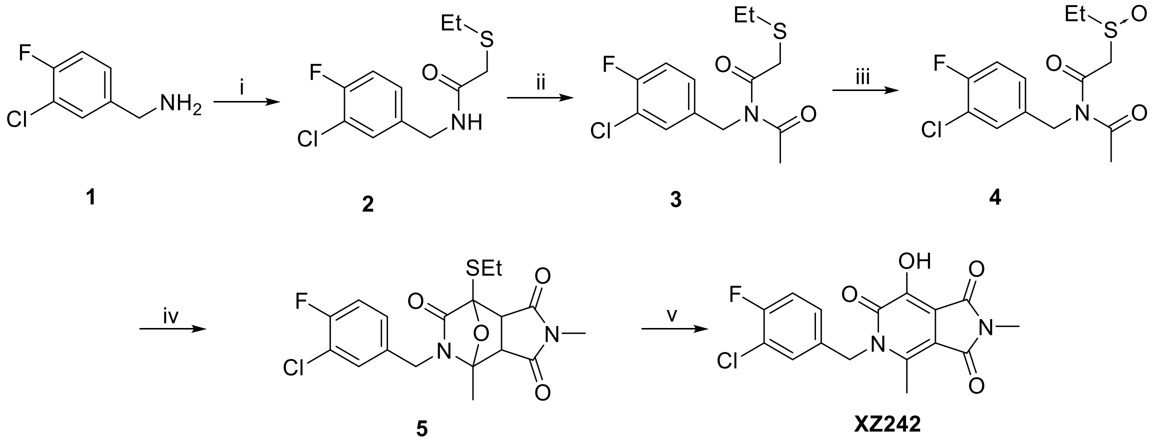

Supplementary Figure 1 | Synthesis of pyridinone XZ242. *Reagents and conditions*: i) $EtSCH_2CO_2H$, $(COCl)_2$, 61%; ii) AcCl, DIPEA, benzene, 80 ºC, 75%; iii) $NaIO_4$, $MeOH/H_2O$, 71%; iv) *p*TsOH, Ac_2O, *N*-Me-maleimide, toluene, 110 ºC, 58%; iv) BF_3-OEt_2, $(CH_2Cl)_2$, rt, 16%.

N-(3-Chloro-4-fluorobenzyl)-2-(ethylthio)acetamide (2).
To 2-ethylthioacetic acid (12.7 mL 157 mmol) in anhydrous DCM (30 mL) was added DMF (0.2 mL) followed by oxalyl chloride (40 mL, 471 mmol), dropwise at 0 ºC, and the mixture was stirred at rt (2 h). The mixture was concentrated under reduced pressure and then added dropwise to a solution of (3-chloro-4-fluorophenyl)methanamine (**1**, 25 g, 157 mmol) in benzene (20 mL) with triethylamine (43.65 mL, 314 mmol) at 0 ºC, and the resultant solution was allowed to come to ambient temperature with stirring (16 h). The mixture was partitioned between EtOAc

and H_2O, and the organic phase was washed by brine, dried by sodium sulfate, filtered and concentrated. the remaining residue was purified by silica gel column chromatography to provide the title compound (**2**, 24.9 g) as a colorless oil (61% yield). ^{1}H NMR (400 MHz, $CDCl_3$) δ 7.28 (dd, *J* = 6.9, 2.1 Hz, 1H), 7.13 – 7.09 (m, 1H), 7.04 (t, *J* = 8.6 Hz, 1H), 4.37 (d, *J* = 6.1 Hz, 2H), 3.22 (s, 2H), 2.50 (q, *J* = 7.4 Hz, 2H), 1.20 (t, *J* = 7.4 Hz, 3H).

N-Acetyl-N-(3-chloro-4-fluorobenzyl)-2-(ethylthio)acetamide (3).
Acetyl chloride (1.72 mL, 242 mmol) was added to amide *N*-(3-chloro-4-fluorobenzyl)-2-(ethylthio)acetamide (**2**, 3.16 g, 121 mmol), in benzene (25 mL), DIPEA (4.22 mL, 242 mmol) was carefully added. The resultant solution was heated and stirred (80 °C, 16 h). The reaction mixture was cooled to rt and extracted by EtOAc, the organic phase was washed by brine, dried by sodium sulfate, filtered and concentrated. The residue was purified by silica gel column chromatography. The title compound (**3**, 2.75 g) was afforded as a colorless oil (yield 75%). ^{1}H NMR (400 MHz, $CDCl_3$) δ 7.22 – 7.18 (m, 1H), 7.09 – 7.01 (m, 2H), 4.90 (s, 2H), 3.67 (s, 2H), 2.54 (q, *J* = 7.3 Hz, 2H), 2.33 (s, 3H), 1.21 (t, *J* = 7.4 Hz, 3H). ^{13}C NMR (101 MHz, $CDCl_3$) δ 173.00, 172.21, 157.40 (d, *J* = 249.1 Hz), 133.83 (d, *J* = 3.9 Hz), 128.36, 125.90 (d, *J* = 7.2 Hz), 121.58 (d, *J* = 18.1 Hz), 116.95 (d, *J* = 21.4 Hz), 46.52, 37.44, 26.20, 26.01, 14.19. ESI-MS m/z: 304.10 (MH^+).

N-Acetyl-N-(3-chloro-4-fluorobenzyl)-2-(ethylsulfinyl)acetamide (4).
The solution of *N*-acetyl-*N*-(3-chloro-4-fluorobenzyl)-2-(ethylthio)acetamide (**3**, 5.06 g, 167 mmol) in MeOH (10 mL) was added dropwise to a solution of sodium periodate (5.56 g, 26 mmol) in MeOH ⁄ H_2O (24 mL, 2:1), and the mixture was stirred at rt (2 h) and then extacted by chloroform, and the organic extracts were dried by sodium sulfate, filtered and taken to dryness to afford the title compound (**4**, 3.78 g) as a colorless oil (yield 71%). This was either used directly or purified by silica gel column chromatography. ^{1}H NMR (400 MHz, $CDCl_3$) δ 7.20 (dd, *J* = 6.8, 2.0 Hz, 1H), 7.10 – 7.01 (m, 2H), 4.90 (q, *J* = 16.8 Hz, 2H), 4.37 (d, *J* = 14.5 Hz, 1H), 4.08 (d, *J* = 14.4 Hz, 1H), 2.93 – 2.75 (m, 2H), 2.28 (s, 3H), 1.32 (t, *J* = 7.5 Hz, 3H). ESI-MS m/z: 320.10 (MH^+).

5-(3-Chloro-4-fluorobenzyl)-7-(ethylthio)-2,4-dimethyltetrahydro-1H-4,7-epoxypyrrolo[3,4-c]pyridine-1,3,6(2H,3aH)-trione (5).
A solution of 3-(3-chloro-4-fluorobenzyl)-5-(ethylthio)-2-methyl-4-oxo-4,5-dihydro-3*H*-oxazol-1-ium-5-ide (**4**, 529 mg, 1.7 mmol) in toluene (4.0 mL) was added dropwise to a solution of 1-methyl-1*H*-pyrrole-2,5-dione (184 mg, 1.65 mmol), acetyl anhydride (1.6 mL, 16.5 mmol) and pTsOH (2 mg) in toluene (4.0 mL). The solution was heated and stirred (110 °C, 2 h). The mixture was cooled to rt and purified by silica gel chromatography. The title compound (**5**, 397 mg) was aforded as a colorless oil (yield 58%). ^{1}H NMR (400 MHz, $CDCl_3$) δ 7.28 (dd, *J* = 6.8, 1.8 Hz, 1H), 7.13 – 7.05 (m, 2H), 4.38 (s, 2H), 3.14 (d, *J* = 6.7 Hz, 1H), 2.91 (s, 3H), 2.89 (d, *J* = 6.8 Hz, 1H), 2.77 (q, *J* = 7.5 Hz, 2H), 1.68 (s, 3H), 1.23 (t, *J* = 7.5 Hz, 3H). ^{13}C NMR (100 MHz, $CDCl_3$) δ 172.53, 171.30, 169.69, 157.82 (d, *J* = 249.5 Hz), 132.99 (d, *J* = 4.6 Hz), 129.94, 127.50 (d, *J* = 6.8 Hz), 121.75 (d, *J* = 17.6 Hz), 117.29 (d, *J* = 21.3 Hz), 95.13, 94.48, 54.82, 49.56, 42.92, 25.42, 24.20, 15.77, 14.56. ESI-MS m/z: 413.10 (MH^+).

5-(3-Chloro-4-fluorobenzyl)-7-hydroxy-2,4-dimethyl-1H-pyrrolo[3,4-c]pyridine-1,3,6(2H,5H)-trione (XZ242).

5-(3-Chloro-4-fluorobenzyl)-7-(ethylthio)-2,4-dimethyltetrahydro-1*H*-4,7-epoxypyrrolo[3,4-*c*]pyridine-1,3,6(2*H*,3a*H*)-trione (**5**, 227 mg, 0.55 mmol) was dissolved in 1,2-dichloroethane (6.0 mL). Boron trifluride diethyl etherate (0.345 ml, 2.75 mmol) was added dorpwose at rt. The reaction was heated and stirred (50°C, 4 h). The reaction mixture was quenced by water and extracted by chloroform. The organic phase was dried by sodium sulfate, filtered and concentrated. The residue was purified by HPLC. The title compound (**XZ242**, 30 mg) was afforded as a white solid (yield 16%) after purification by preparative HPLC (with a linear gradient of 40 % B to 60 % B over 30 minutes; retention time = 20.2 minutes). ^{1}H NMR (400 MHz, DMSO-d$_6$) δ 11.02 (bs, 1H), 7.39 (dd, *J* = 7.1, 2.2 Hz, 1H), 7.32 (t, *J* = 9.0 Hz, 1H), 7.13 – 7.09 (m, 1H), 5.26 (s, 2H), 2.88 (s, 3H), 2.54 (s, 3H). ^{13}C NMR (100 MHz, DMSO-d$_6$) δ 166.92, 164.99, 161.05, 156.85 (d, *J* = 246.3 Hz), 142.57, 139.03, 134.30 (d, *J* = 3.7 Hz), 129.08, 127.46 (d, *J* = 7.4 Hz), 120.15 (d, *J* = 17.8 Hz), 117.57 (d, *J* = 20.8 Hz), 112.95, 106.23, 46.57, 24.16, 14.51. ESI-MS m/z: 351.00 (MH$^+$). HRMS calcd. for $C_{16}H_{13}N_2O_4FCl$ [MH$^+$], 351.0542; found, 351.0538.

II. Preparation of 9-(3-Chloro-4-fluorobenzyl)-2-ethyl-7-hydroxy-3,4-dihydropyrrolo[3',4':3,4]pyrido[1,2-*a*]pyrazine-1,6,8,10(2*H*,9*H*)-tetraone (XZ256).

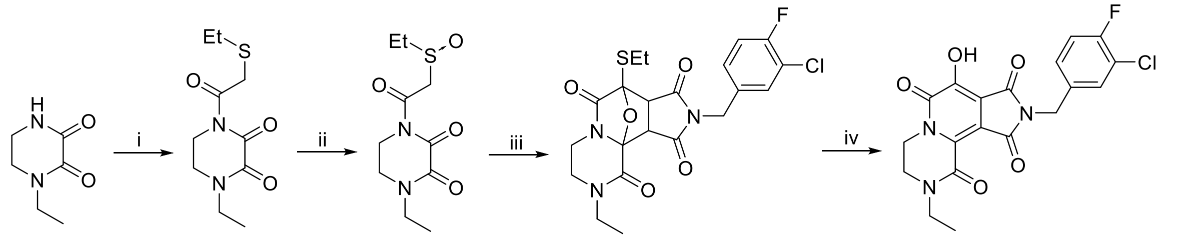

6 **7** **8** **9** **XZ256**

Supplementary Figure 2 | Synthesis of pyridinone XZ256. *Reagents and conditions*: i) $EtSCH_2CO_2H$, $(COCl)_2$, benzene, 80 °C, 60%; ii) *m*CPBA, $CHCl_3$; iii) *N*-4'-F-3'-Cl-Benzyl-maleimide, toluene, 100 °C, 34% (two steps); iv) BF_3-OEt_2, rt, 44%.

1-Ethyl-4-(2-(ethylthio)acetyl)piperazine-2,3-dione (7).

To 2-ethylthioacetic acid (2.22 mL, 27.4 mmol) in anhydrous DCM (10 mL) was added DMF (0.1 mL) followed by oxalyl chloride (2.8 ml, 31.7 mmol), dropwise at 0 °C, and the mixture was stirred (rt, 2 h). The mixture was concentrated under reduced pressure and then added dropwise to a solution of 1-ethylpiperazine (**6**, 3.0 g, 21 mmol) in benzene (20 mL). Triethylamine (4.4 mL) was added carefully. The reaction was heated and stirred (80 °C, 2 h). The reaction mixture was cooled to rt, extracted by DCM and washed by brine. The organic phase was dried by anhydrous sodium sulfate, filtered and concentrated. The residue was purified by silica gel chromatography. The title compound (**7**, 3.14 g) was fforded as a yellow solid (yield 60%). ^{1}H NMR (400 MHz, CD_3OD) δ 4.03 – 3.99 (m, 2H), 3.88 (d, *J* = 0.8 Hz, 2H), 3.63 – 3.59 (m, 2H), 3.50 (qd, *J* = 7.2, 0.8 Hz, 2H), 2.55 (qd, *J* = 7.4, 0.8 Hz, 2H), 1.19 (dtd, *J* = 10.9, 7.3, 0.8 Hz, 6H). ^{13}C NMR (101 MHz, $CDCl_3$) δ 171.34, 157.46, 155.90, 43.71, 42.69, 40.83, 38.04, 26.10, 14.31, 12.18. ESI-MS m/z: 245.1 (MH$^+$).

9-(3-Chloro-4-fluorobenzyl)-2-ethyl-7-(ethylthio)tetrahydro-1H-7,10b-epoxypyrrolo[3',4':3,4]pyrido[1,2-a]pyrazine-1,6,8,10(2H,9H,10aH)-tetraone (9).

1-Ethyl-4-(2-(ethylthio)acetyl)piperazine-2,3-dione (**7**, 235 mg, 0.962 mmol) was dissolved in chloroform (10 mL). *m*CPBA (216 mg, 0.962 mmol) was added at -78 °C

for 15 min. The reaction mixture was warmed to 0 °C and filtered. The filterate was concentrated. Compound1-ethyl-4-(2-(ethylsulfinyl)acetyl)piperazine-2,3-dione (**8**, 250 mg) was afforded. [^{1}H NMR (400 MHz, $CDCl_3$) δ 4.50 (d, *J* = 14.7 Hz, 1H), 4.22 (d, *J* = 14.7 Hz, 1H), 4.07 – 4.03 (m, 2H), 3.60 – 3.55 (m, 2H), 3.51 (q, *J* = 7.3 Hz, 2H), 3.00 – 2.88 (m, 2H), 1.34 (t, *J* = 7.5 Hz, 3H), 1.17 (t, *J* = 7.2 Hz, 3H)] The solution of the crude residue 1-ethyl-4-(2-(ethylsulfinyl)acetyl)piperazine-2,3-dione (**8**, 250 mg) in toluene (3.0 mL) was added dropwise to the solution of 1-(3-chloro-4-fluorobenzyl)-1H-pyrrole-2,5-dione (230 mg, 0.962 mmol), acetic anhydride (0.9 mL, 9.62 mmol) and pTsOH (2 mg) in toluene (3.0 mL) at 100 °C. The reaction was heated and stirred (100 °C, 2 h). The result mixture was cooled to rt and purified by silica gel column chromatography. The title compound (**9**, 157 mg) was afforded as a colorless oil (yield 34 % for two steps). ^{1}H NMR (400 MHz, $CDCl_3$) δ 7.31 (dd, *J* = 6.9, 2.0 Hz, 1H), 7.18 – 7.14 (m, 1H), 7.05 (t, *J* = 8.4 Hz, 1H), 4.61 (d, *J* = 14.1 Hz, 1H), 4.30 (d, *J* = 14.1 Hz, 1H), 4.23 (d, *J* = 8.5 Hz, 1H), 3.67 (d, *J* = 8.5 Hz, 1H), 3.55-3.46 (m, 2H), 3.44-3.38 (m, 2H), 3.26-3.21 (m, 1H), 2.83 – 2.77 (m, 2H), 2.63 – 2.56 (m, 1H), 1.23 (dt, *J* = 7.5, 0.7 Hz, 3H), 1.16 (t, *J* = 7.1 Hz, 3H). ESI-MS m/z: 482.0 (MH^+).

9-(3-Chloro-4-fluorobenzyl)-2-ethyl-7-hydroxy-3,4-dihydropyrrolo[3',4':3,4]pyrido[1,2-a]pyrazine-1,6,8,10(2H,9H)-tetraone (XZ256).
9-(3-Chloro-4-fluorobenzyl)-2-ethyl-7-(ethylthio)tetrahydro-1*H*-7,10b-epoxypyrrolo[3',4':3,4]pyrido[1,2-*a*]pyrazine-1,6,8,10(2*H*,9*H*,10a*H*)-tetraone (**9**, 74.7 mg, 0.155 mmol) was dissolved in DCM (1.0 mL). Boron trifluride etherate (97 µL, 0.775 mmol) was added. The mixture was stirred (rt, 16 h). The result mixture was concenterated. The title compound (**XZ256**, 29 mg) was afforded as a white solid (yield 44%) after purification by preparative HPLC (with a linear gradient of 40 % B to 60 % B over 30 minutes; retention time = 15.6 minutes). ^{1}H NMR (400 MHz, DMSO-d_6) δ 7.44 (dd, *J* = 7.0, 2.2 Hz, 1H), 7.33 (t, *J* = 9.0 Hz, 1H), 7.26 – 7.21 (m, 1H), 4.63 (s, 2H), 4.13 – 4.08 (m, 2H), 3.62 – 3.56 (m, 2H), 3.44 (dd, *J* = 14.5, 7.4 Hz, 3H), 1.07 (t, *J* = 7.0 Hz, 3H). ^{13}C NMR (125 MHz, DMSO-d_6) δ 164.43, 162.42, 157.97, 156.99 (d, *J* = 246.1 Hz), 155.20, 146.46, 135.04 (d, *J* = 3.8 Hz), 130.20, 129.48, 128.87 (d, *J* = 7.6 Hz), 119.84 (d, *J* = 17.8 Hz), 117.47 (d, *J* = 21.0 Hz), 113.42, 110.60, 43.22, 43.07, 41.60, 40.36, 12.91. MALDI-MS m/z: 420.02 (MH^+). HRMS calcd. for $C_{19}H_{16}N_3O_5FCl$ [MH^+], 420.0757; found, 420.0754.

III. Preparation of 4-Amino-*N*-(2,4-difluorobenzyl)-1-hydroxy-2-oxo-6-phenyl-1,2-dihydro-1,8-naphthyridine-3-carboxamide (XZ420).

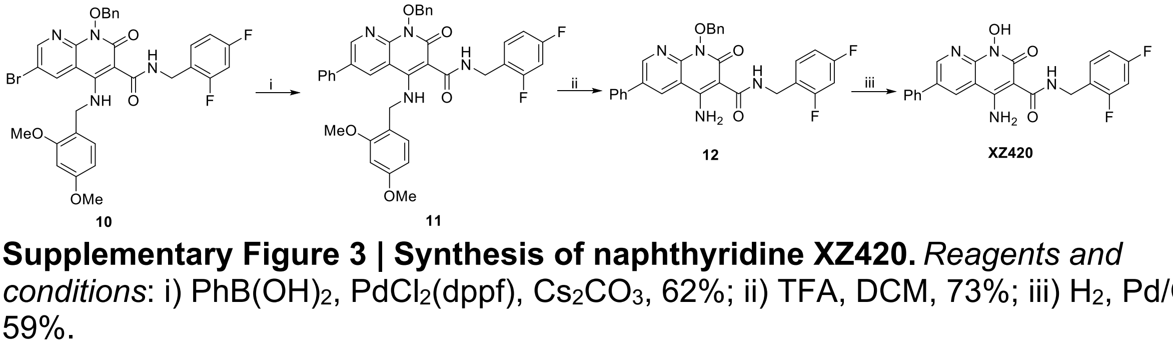

Supplementary Figure 3 | Synthesis of naphthyridine XZ420. *Reagents and conditions*: i) $PhB(OH)_2$, $PdCl_2(dppf)$, Cs_2CO_3, 62%; ii) TFA, DCM, 73%; iii) H_2, Pd/C, 59%.

1-(Benzyloxy)-N-(2,4-difluorobenzyl)-4-((2,4-dimethoxybenzyl)amino)-2-oxo-6-phenyl-1,2-dihydro-1,8-naphthyridine-3-carboxamide (11).

A mixture of 1-(benzyloxy)-6-bromo-*N*-(2,4-difluorobenzyl)-4-((2,4-dimethoxybenzyl)amino)-2-oxo-1,2-dihydro-1,8-naphthyridine-3-carboxamide (**10**, 147 mg, 0.22 mmol)[1], cesium carbonate (288 mg, 0.88 mmol), phenylboronic acid (108 mg, 0.88 mmol) and [1,1'-bis(diphenylphosphino)ferrocene]dichloropalladium(**II**) (8 mg, 0.011 mmol) in toluene (2.0 mL) was flushed with argon and heated (100 °C, 8 h). The reaction mixture was cooled to rt and purified by silica gel column chromatography. The title compound (**11**, 89 mg) was afforded as a white solid (yield 62%). ^{1}H NMR (400 MHz, $CDCl_3$) δ 12.17 (t, *J* = 6.3 Hz, 1H), 10.79 (t, *J* = 5.7 Hz, 1H), 8.92 – 8.79 (m, 1H), 8.40 (d, *J* = 2.1 Hz, 1H), 7.68 (d, *J* = 7.4 Hz, 2H), 7.39 - 7.27 (m, 10H), 6.85 – 6.75 (m, 2H), 6.47 - 6.45 (m, 2H), 5.27 (s, 2H), 4.77 (d, *J* = 6.5 Hz, 2H), 4.60 (d, *J* = 5.7 Hz, 2H), 3.79 (s, 3H), 3.63 (s, 3H). ESI-MS m/z: 663.2 (MH^+).

4-Amino-1-(benzyloxy)-N-(2,4-difluorobenzyl)-2-oxo-6-phenyl-1,2-dihydro-1,8-naphthyridine-3-carboxamide (12).
1-(Benzyloxy)-*N*-(2,4-difluorobenzyl)-4-((2,4-dimethoxybenzyl)amino)-2-oxo-6-phenyl-1,2-dihydro-1,8-naphthyridine-3-carboxamide (**11**, 89 mg, 0.135 mmol) was dissolved in DCM (2.0 mL). TFA (2.0 mL) was added at rt. The solvent was evaporated and the residue was purified by silica gel column chromatography. The title compound (**12**, 51 mg) was afforded as a white solid (yield 73 %). ^{1}H NMR (500 MHz, $CDCl_3$) δ 10.65 (t, *J* = 5.9 Hz, 1H), 8.96 (dd, *J* = 2.1, 0.8 Hz, 1H), 8.22 (dd, *J* = 2.1, 0.8 Hz, 1H), 7.69 – 7.66 (m, 2H), 7.60 (d, *J* = 7.1 Hz, 2H), 7.48 (t, *J* = 7.7 Hz, 2H), 7.43 – 7.34 (m, 5H), 6.86 – 6.77 (m, 2H), 5.29 (s, 2H), 4.62 (d, *J* = 5.8 Hz, 2H). ESI-MS m/z: 513.1 (MH^+).

4-Amino-N-(2,4-difluorobenzyl)-1-hydroxy-2-oxo-6-phenyl-1,2-dihydro-1,8-naphthyridine-3-carboxamide (XZ420).
4-Amino-1-(benzyloxy)-*N*-(2,4-difluorobenzyl)-2-oxo-6-phenyl-1,2-dihydro-1,8-naphthyridine-3-carboxamide (**12**, 61 mg, 0.12 mmol) was dissolved in MeOH (10 mL). Pd/C (28 mg, 0.12 mmol) was added. After degassed, the reaction was stirred under hydrogen (rt, 1.5 h). The mixture was filtered and concentrated. The title compound (**XZ420**, 30 mg) was afforded as a yellow solid (yield 59 %) after purification by preparative HPLC (with a linear gradient of 30 % B to 70 % B over 30 minutes; retention time = 27.3 minutes). ^{1}H NMR (500 MHz, DMSO-d_6) δ 10.67 (t, *J* = 5.8 Hz, 1H), 9.08 20 (d, *J* = 2.1 Hz, 1H), 9.01 (d, *J* = 2.1 Hz, 1H), 7.87 (dd, *J* = 8.2, 1.0 Hz, 2H), 7.55 (t, *J* = 7.7 Hz, 2H), 7.46- 7.41 (m, 2H), 7.27 - 7.22 (m, 1H), 7.08 (td, *J* = 8.6, 2.5 Hz, 1H), 4.54 (d, *J* = 5.8 Hz, 2H). ^{13}C NMR (125 MHz, DMSO-d_6) δ 169.07, 161.87 (dd, *J* = 245.2, 12.2 Hz), 161.11, 160.67 (dd, *J* = 247.1, 12.3 Hz), 155.68, 151.42, 147.25, 136.43, 131.58, 131.26 (dd, *J* = 9.8, 6.1 Hz), 130.62, 129.60 (2C), 128.59, 127.23 (2C), 123.11 (dd, *J* = 15.2, 3.6 Hz), 111.83 (dd, *J* = 20.9, 3.6 Hz), 108.93, 104.27 (t, *J* = 25.8 Hz), 92.61, 36.12 (d, J = 3.6 Hz). ESI-MS m/z: 423.1 (MH^+). HRMS calcd $C_{22}H_{17}F_2N_4O_3$ [MH^+], 423.1263; found, 423.1243.

Statistical analysis and significance

Statistical analysis was conducted in Prism 9. Where appropriate, statistical significance was calculated with a parametric t-test with Welch's correction.

Sample size for the cryo-EM studies was determined by the availability of microscope time and to ensure unambiguous modeling of the structures. Sample sizes of the cryo-EM data sets are included in the Supplementary Table 6 and in the processing flow chart in Supplementary Figure 9.

Supplementary figures

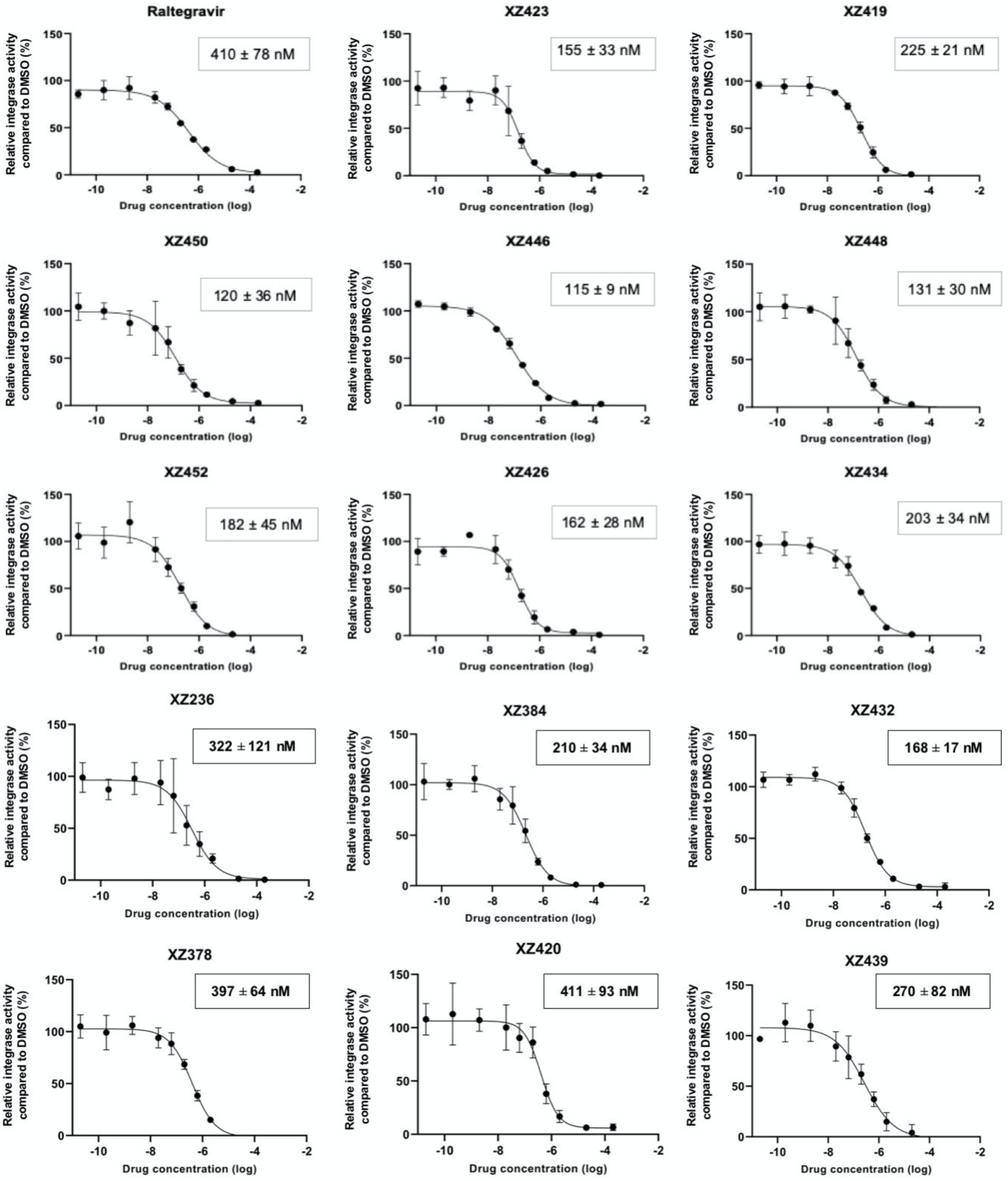

Supplementary Figure 4 | *In vitro* integrase strand-transfer activity assays were used to determine the dose-response curve and the IC_{50} for each drug. Averages and standard deviations are shown for n = three biologically independent biological experiments. The value in brackets is the mean IC_{50} ± standard deviation.

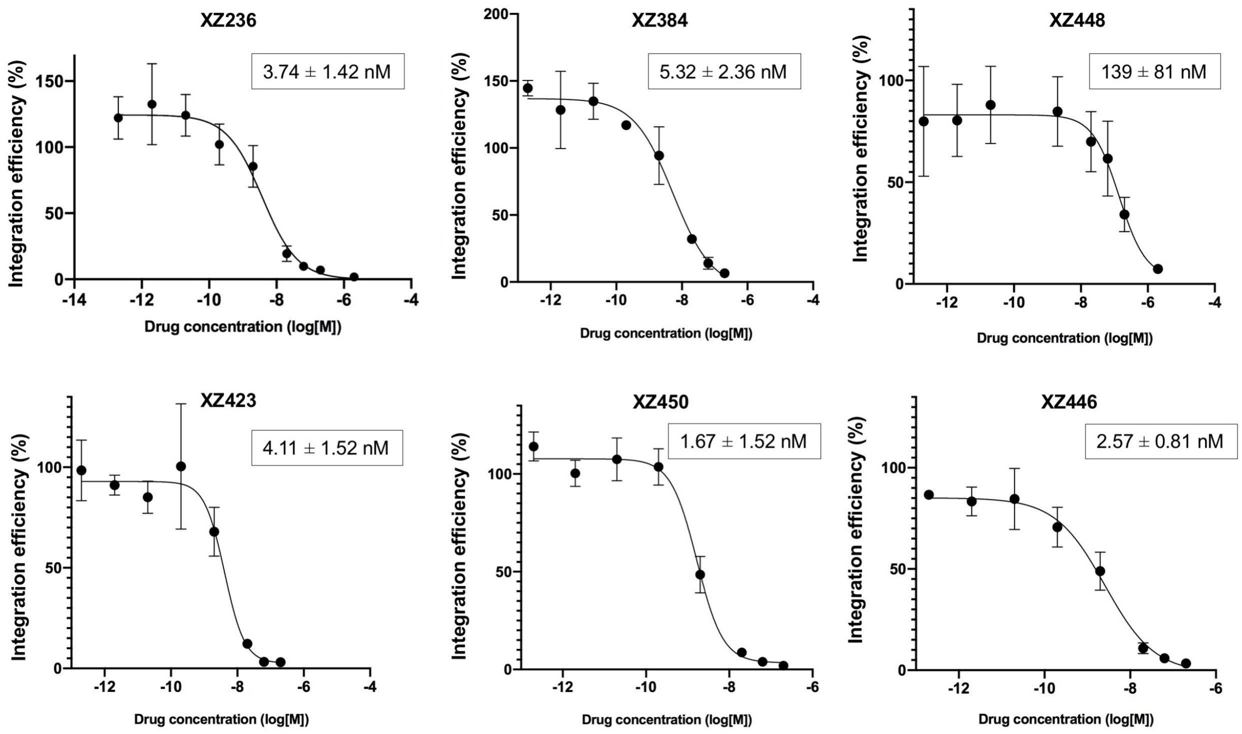

Supplementary Figure 5 | *In cellulo* assays performed to assess the efficacy of a panel of novel INSTIs on HTLV-1 infectivity in a cell-based infection model. Averages and standard deviations are shown for n = three biologically independent experiments. The value in brackets is the mean EC_{50} ± standard deviation.

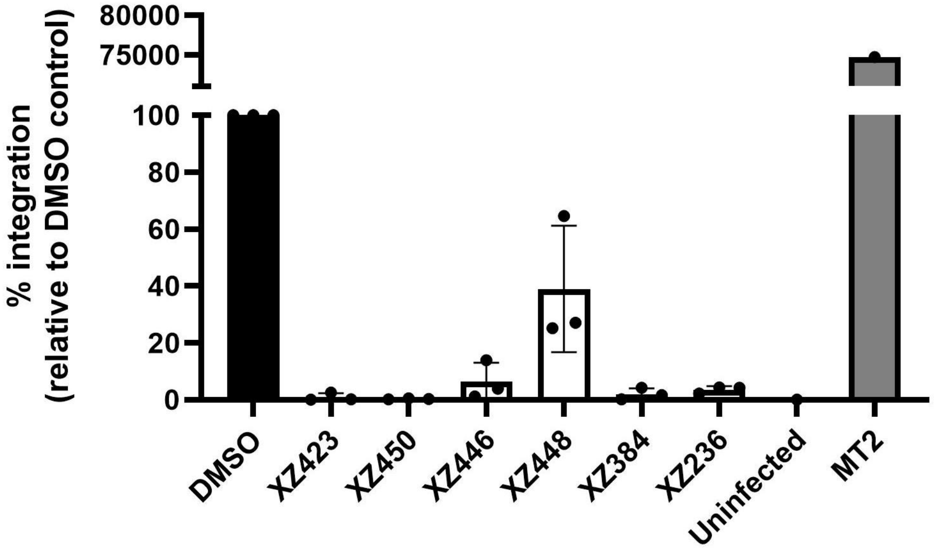

Supplementary Figure 6 | Integrated provirus was quantified by Alu-qPCR. Values were normalized to GAPDH. Uninfected represents uninfected Jurkat cells, acting as negative control. Condition without any drug present (DMSO) was set as 100%. Mean values and standard deviations are shown for n = three biologically independent experiments. *P*-values were calculated with respect to the DMSO control using the unpaired t-test with Welch's correction in Prism 9. *P*-values are two-sided. As compared to raltegravir control. XZ423, $p < 0.0001$; XZ384, $p = 0.0001$; XZ450, p

< 0.0001; XZ446, $p = 0.0017$; XZ434, $p < 0.0001$; XZ448, $p = 0.0414$; XZ236, $p <$ 0.0001. Source data are provided as a Source Data File.

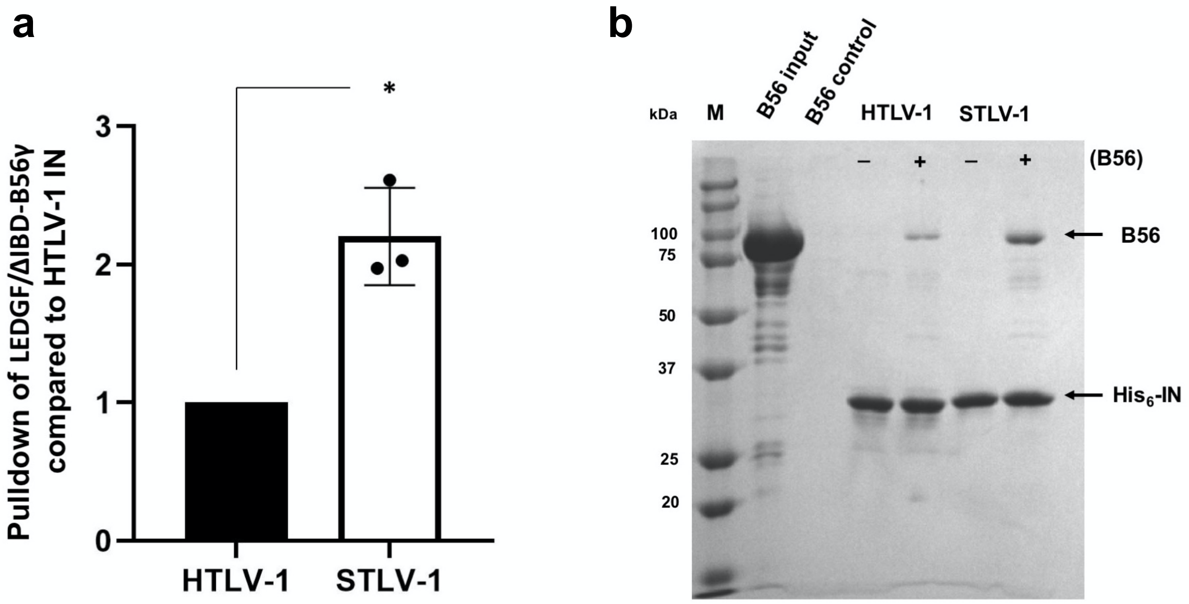

Supplementary Figure 7 | Pull-down assays of LEDGF/ΔIBD-B56γ by His$_6$-tagged HTLV-1 and STLV-1 INs. a, Densitometry quantification of pull-down assays as shown in panel b. Values are normalised to HTLV-1 IN. Mean values and standard deviations are shown for n = three biologically independent experiments. The *p*-value was calculated using the unpaired t-test with Welch's correction in Prism 9. The *p*-value is two-sided. As compared to HTLV-1. STLV-1, $p = 0.0274$. **b**, Representative example of Coomassie brilliant blue stained gel following separation of pull-down assay samples on SDS-PAGE. Migration of His$_6$-IN and LEDGF/ΔIBD-B56 (labelled as B56) are indicated to the right of the gel. Mw marker is indicated on the left of the gel. Source data are provided as a Source Data File.

a

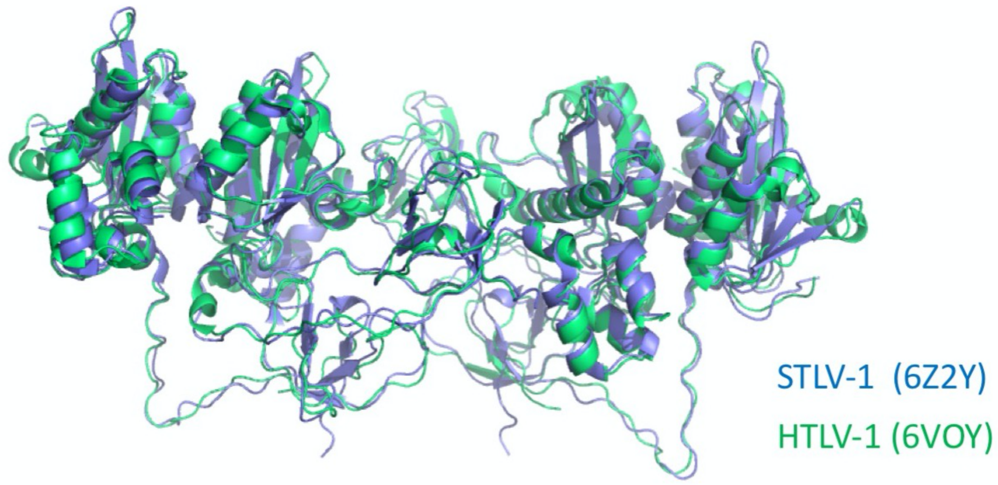

b

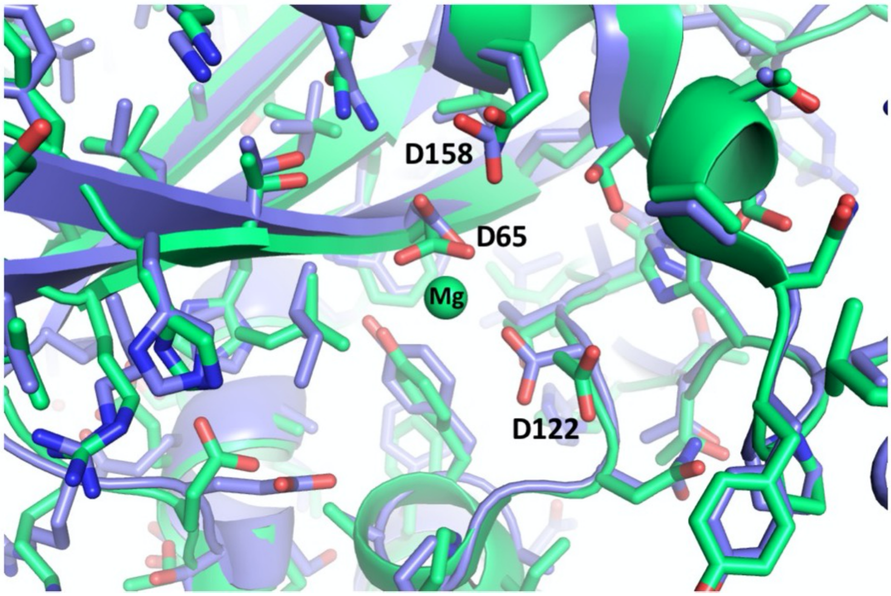

Supplementary Figure 8 | Overlay of cryo-EM structures of STLV-1 (6Z2Y, blue) and HTLV-1 (6VOY, green) intasomes. a, Overlay showing secondary structures of both models reveals near-identical topology between the two models. B56γ and vDNA have been removed from both structures for clarity. RMSD between the two structures is 29.275 Å**. b**, Comparison of side chain conformations in both models. The IN DDE catalytic triad residues are indicated in the STLV-1 structure. Note that only one active site Mg^{2+} is bound by the apo-structures, shown here as a green sphere.

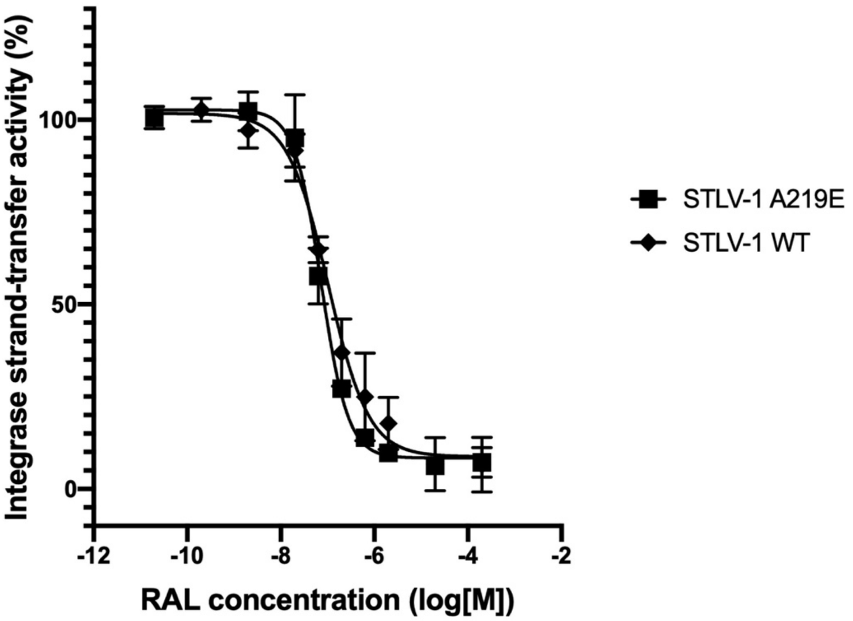

Supplementary Figure 9 | Raltegravir (RAL) dose-response relationship curves for wild type and A219E STLV-1 INs determined in *in vitro* strand-transfer assays. Mean values and standard deviations are shown for n = three biologically independent experiments. Source data are provided as a Source Data File.

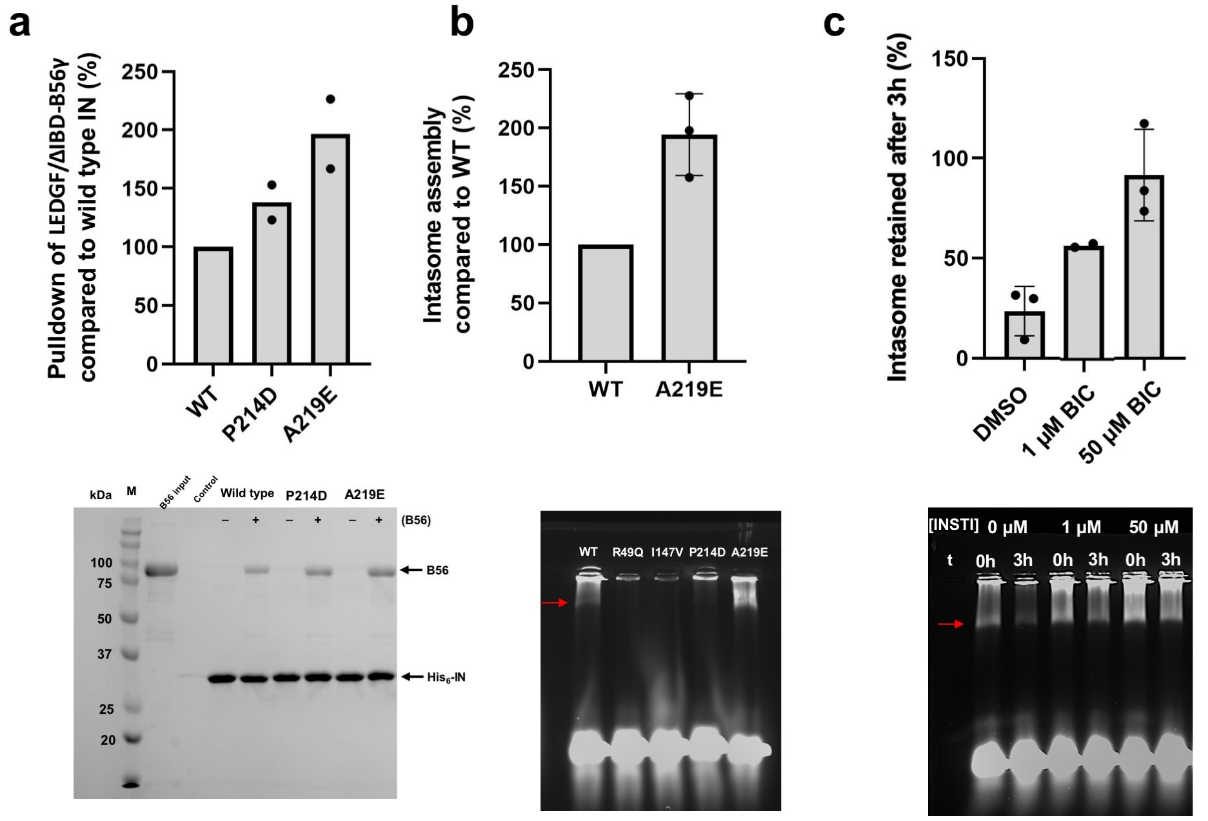

Supplementary Figure 10 | STLV-1 IN A219E binds more B56γ and stabilises the deltaretroviral intasome. a, Pulldown of LEDGF/ΔIBD-B56γ by wild type His_6-STLV-1 IN and its mutants. Mutations introduced in the IN:B56 binding interface can strengthen the interaction. **b**, A219E-mutated STLV-1 IN increases the amount of intasome assembled *in vitro* in an EMSA assay, compared to wild type IN. Values are normalised to wild type STLV-1 IN. **c**, EMSA study of the effect of addition of BIC on the stability (retention) of the assembled STLV-1 intasome after three hours of incubation with the drug. Values shown represent percentage of intasome retained as compared to intasome assayed by EMSA straight after assembly. Averages and standard deviations are shown for two independent biological replicates. Primary data from pull-down assays and EMSA is also included below each panel. The red arrow indicates the intasome band. INSTI, integrase strand transfer inhibitor. Source data are provided as a Source Data File.

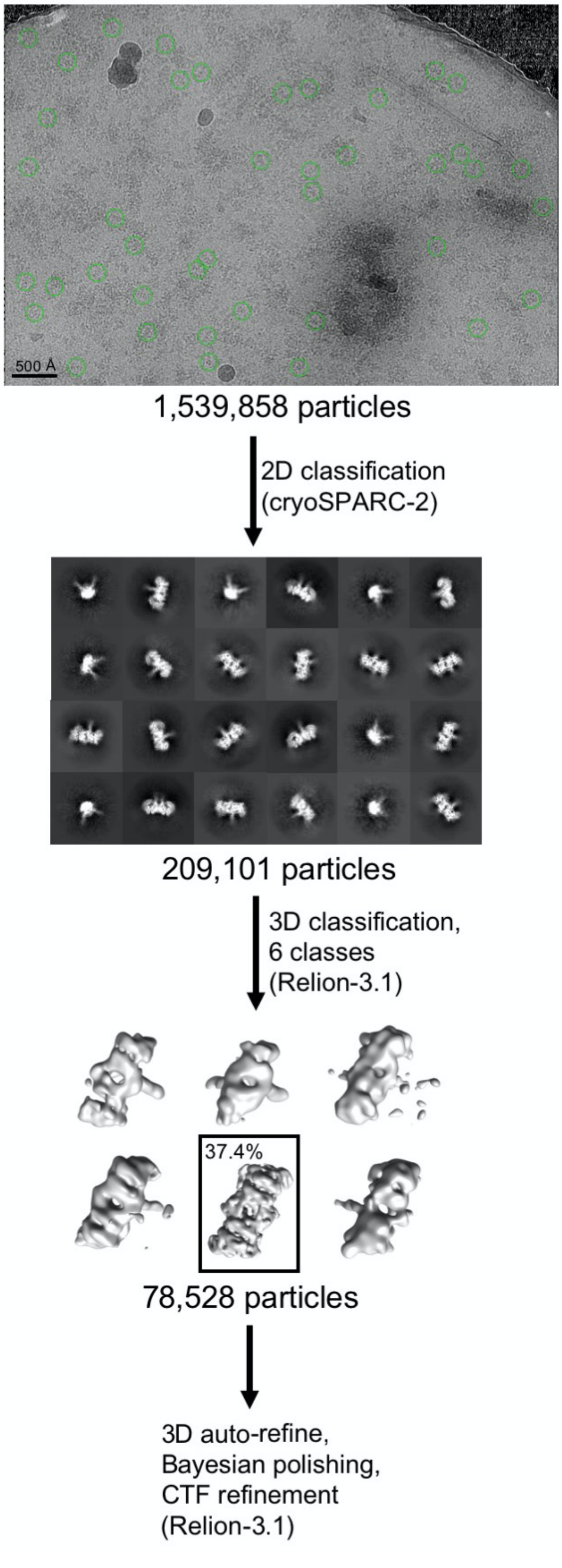
500 Å
1,539,858 particles
2D classification
(cryoSPARC-2)
209,101 particles
3D classification,
6 classes
(Relion-3.1)
37.4%
78,528 particles
3D auto-refine,
Bayesian polishing,
CTF refinement
(Relion-3.1)

Supplementary Figure 11 | Schematic of cryo-EM image processing for the STLV-1 intasome$^{IN(A219E)}$:LEDGF/ΔIBD-B56γ:XZ450 complex. Particles were imaged on graphene oxide-coated grids and analysed according to the workflow above using Cryosparc and Relion software. The workflow was then applied to subsequent RAL and XZ450 datasets. Details are given in Materials and Methods.

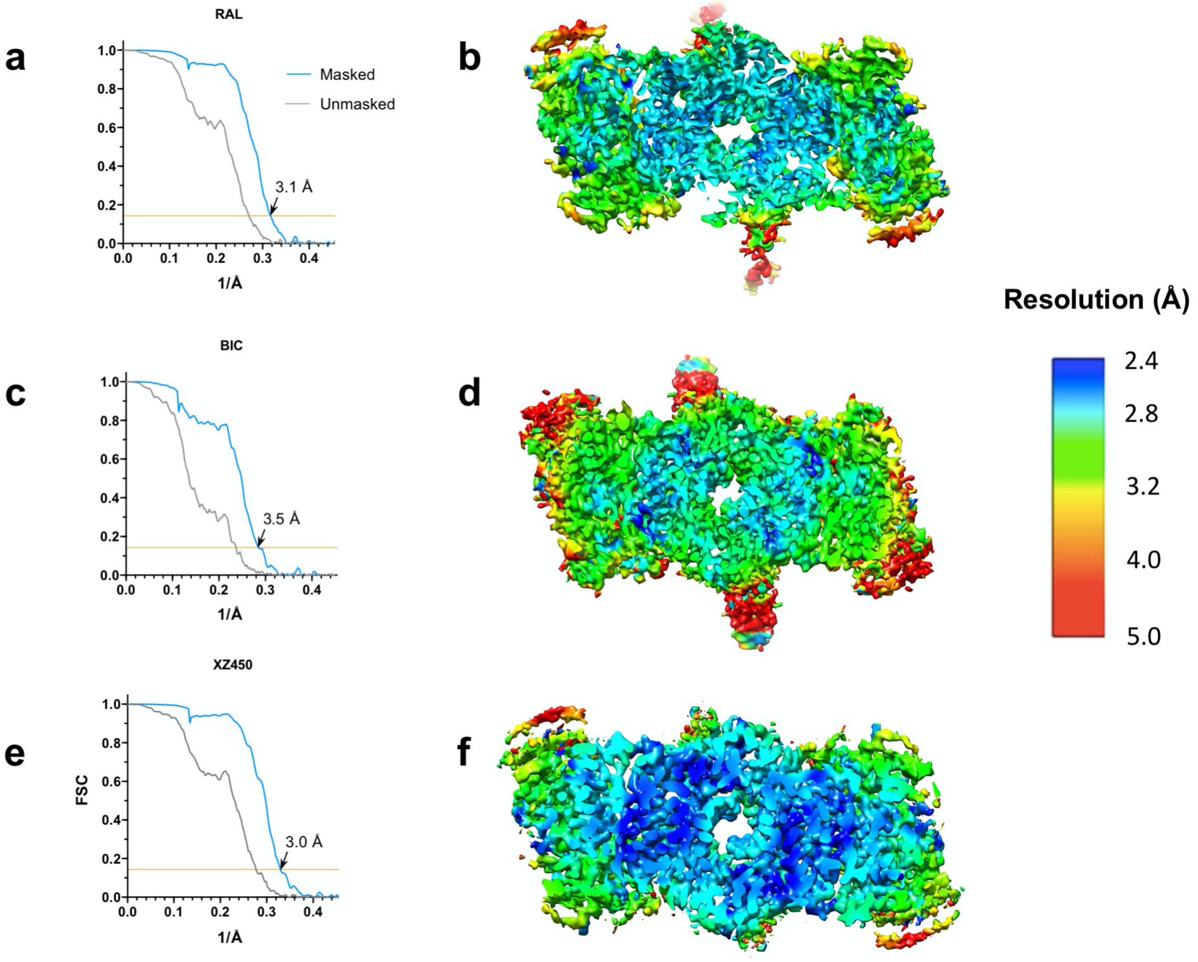

Supplementary Figure 12 | Analysis of the reconstructed cryo-EM maps. Fourier shell correlation (FSC) curves and local resolution maps are shown for the raltegravir (RAL) (**a-b**), bictegravir (BIC) (**c-d**) and XZ450 (**e-f**) datasets. Local resolution of each cryo-EM map is indicated in colour from highest (blue) to lowest (red) overlaid on a cross-section through the map of each intasome structure shown from top view (DNA facing away).

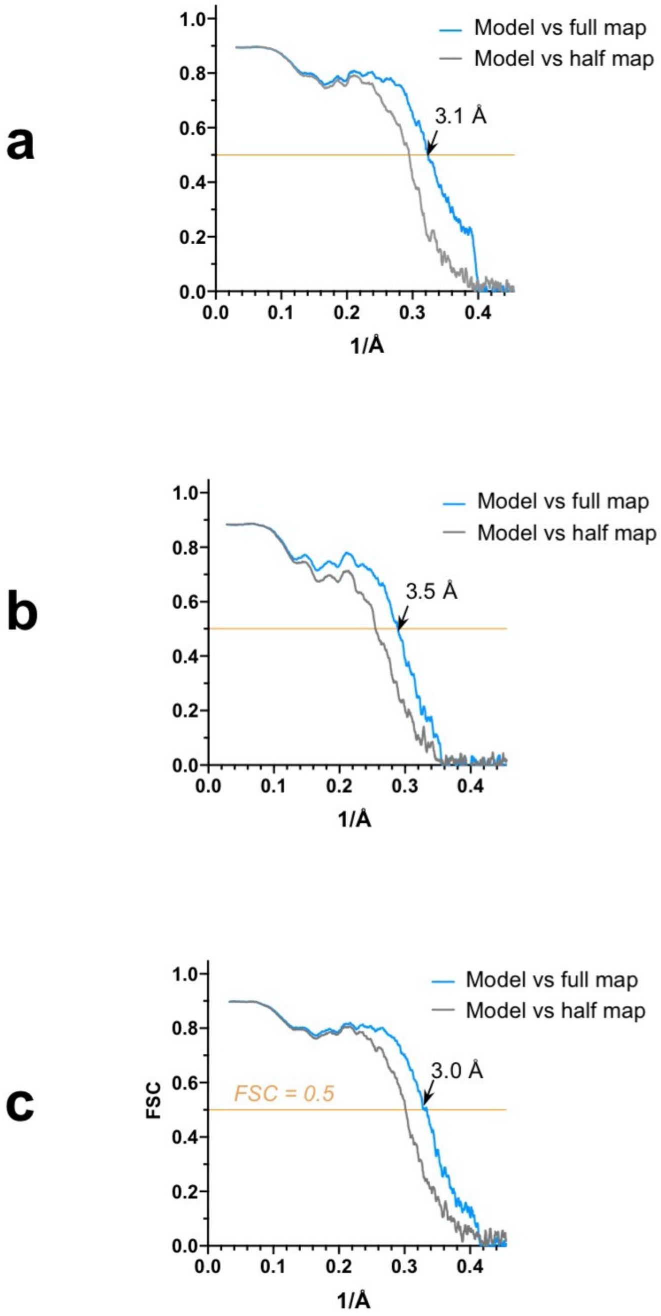

Supplementary Figure 13 | Analysis of the cryo-EM structure model of raltegravir (a), bictegravir (b) and XZ450 (c). Fourier shell correlation (FSC) of the refined models and density-modified full maps (blue lines) or half-maps (grey lines). Horizontal lines (orange) correspond to FSC=0.5.

a

b

c

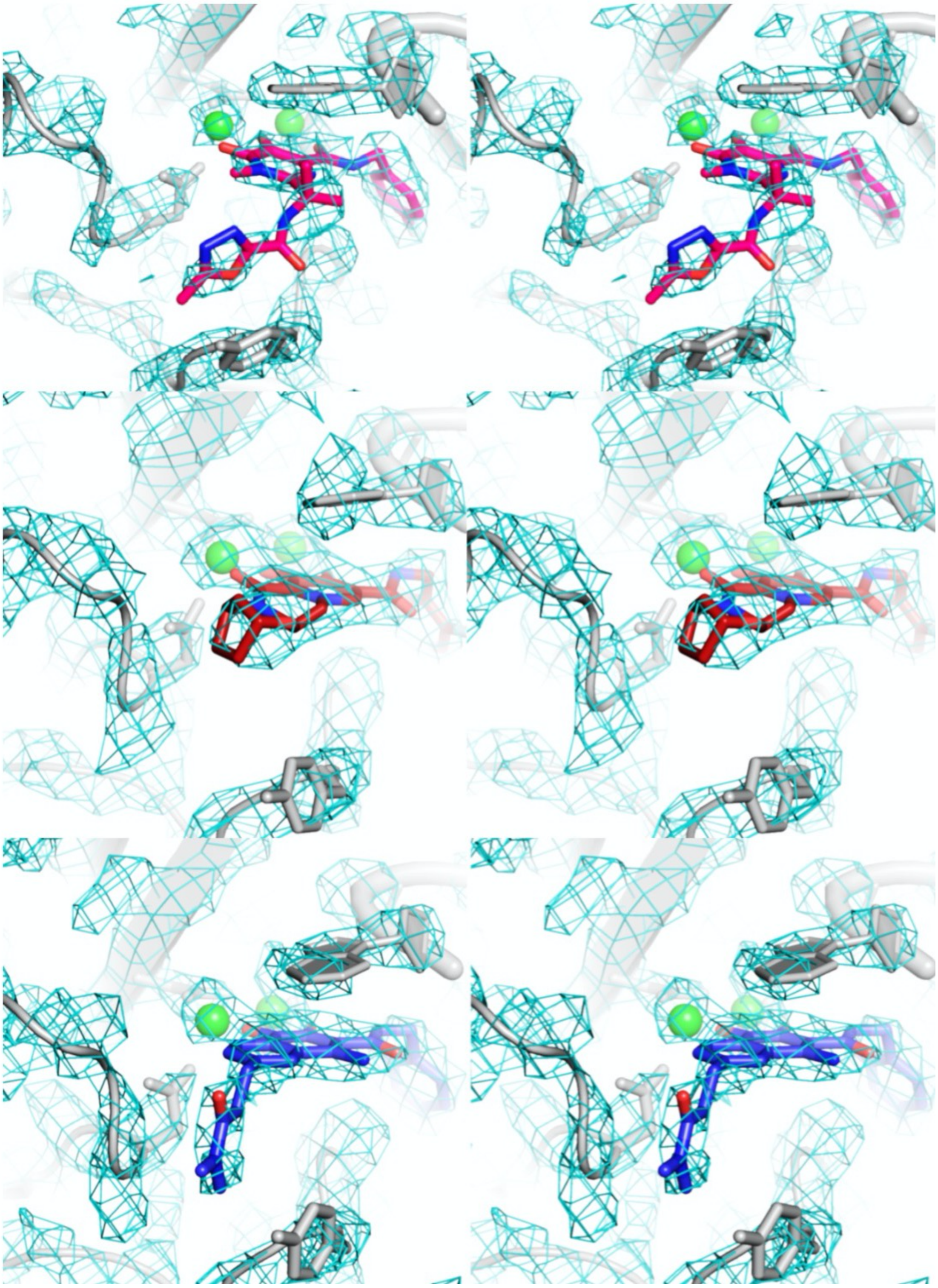

Supplementary Figure 14 | Active sites of STLV intasome:INSTI complexes. a, raltegravir; **b**, bictegravir and **c**, XZ450 compounds are shown in magenta, red and blue, respectively. The nucleoprotein structures to which each of the compounds belong are coloured in grey with the DDE catalytic triad and residue Y149 shown in sticks. Magnesium ions are shown as green spheres. Overlaid on each structure is

their respective cryo-EM map in cyan wireframe. Pairs of panels are presented in wall-eye stereo view.

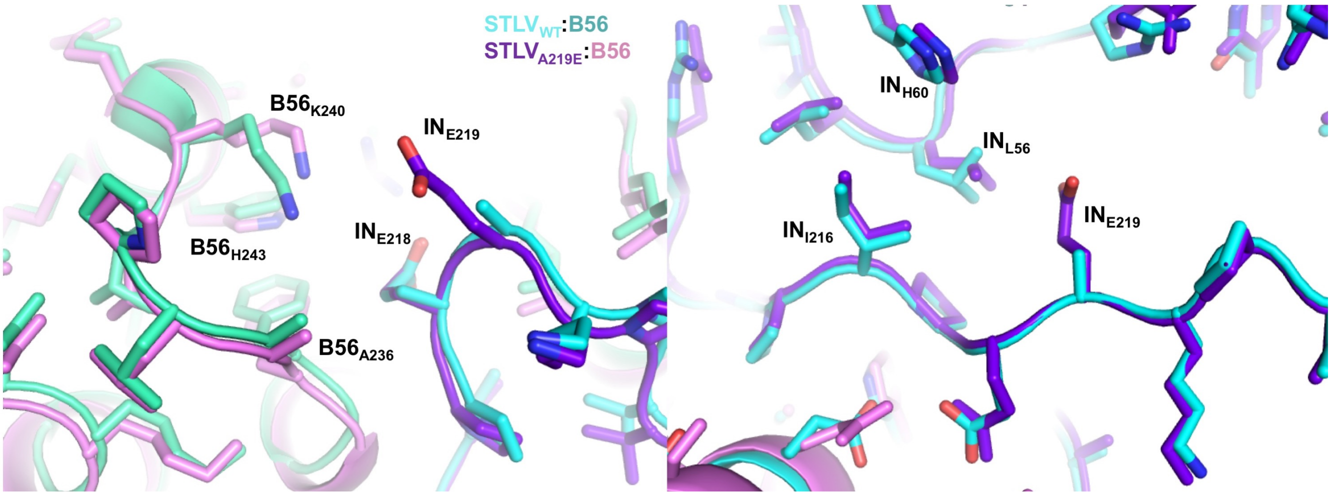

Supplementary Figure 15 | Putative interactions of IN residue E219 in the structure of STLV-1 intasome^{A219E}:B56 in complex with XZ450 overlaid on the apo intasome:B56 structure (PDB ID: 6Z2Y). The IN and B56 chains in the WT intasome structure are coloured cyan and light green respectively, in the A219E intasome structure IN is purple and B56 magenta. E219 is facing the B56 interface in one IN chain (left panel) while it faces the IN CCD domain in the other chain (right panel).

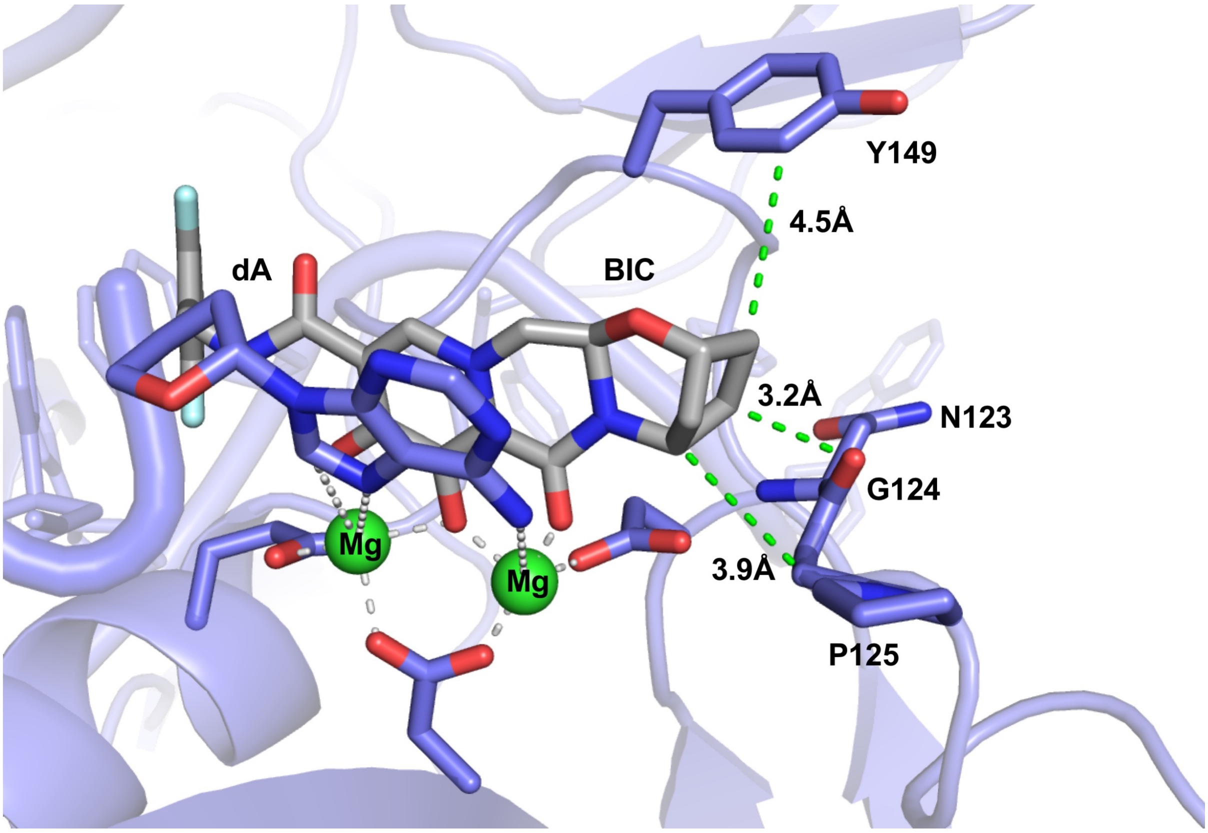

Supplementary Figure 16 | Bictegravir (BIC) is shown bound to the STLV-1 intasome. View of the STLV-1 intasome active site. Contacts between the terminal heterocycle of BIC and residues N123 and G124 of IN (purple) are shown (green dashed lines), with distances indicated. BIC is shown as sticks with carbon atoms in grey. Active site Mg^{2+} ions are shown in green. Coordination of the active site Mg^{2+} ions is shown in grey dashed lines. dA, deoxyadenosine.

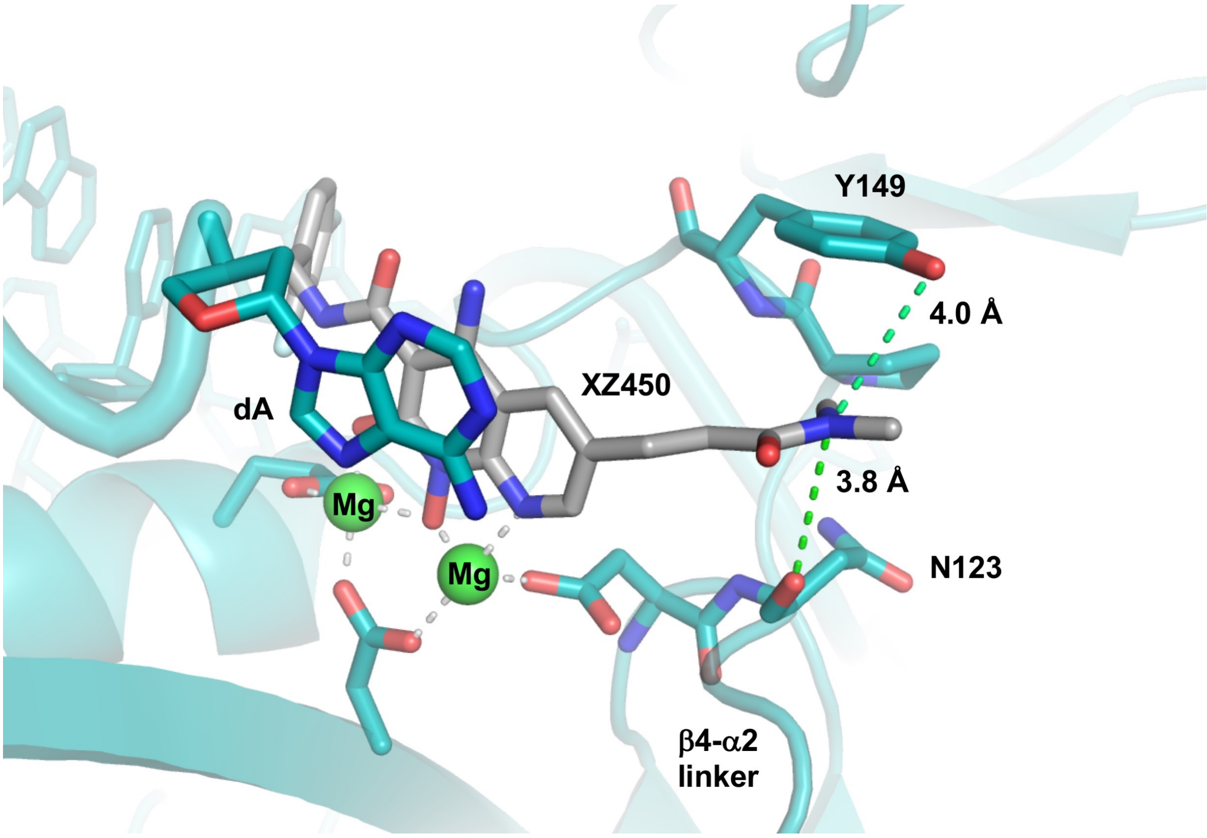

Supplementary Figure 17 | XZ450 is shown bound to the STLV-1 intasome. View of the STLV-1 intasome active site. Contacts between the dimethylaminooxopropyl group and the β4-α2 linker and residue Y149 of STLV-1 IN (cyan) are shown. XZ450 is shown as sticks with carbon atoms in grey. Active site Mg^{2+} ions are shown as green spheres, their coordination in grey dashed lines. dA, deoxyadenosine.

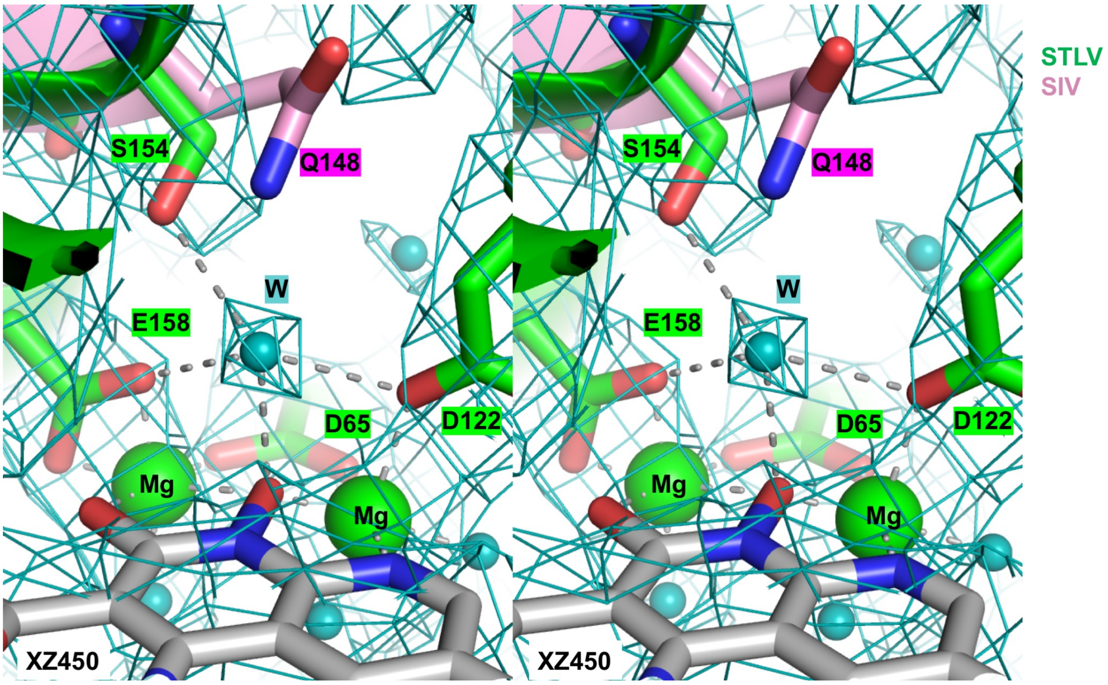

Supplementary Figure 18 | Interactions of the STLV-1 catalytic residues (green) and S154 with a water molecule (W, red sphere) within the active site. SIV IN Q148 (pink) and STLV-1 IN S154 (green) interact with a key water molecule (turquoise sphere, W) that is coordinated by the carboxylates of E158 and D122. The SIV intasome:BIC structure (PDB ID: 6RWM) is overlaid on the STLV intasome:XZ450 cryo-EM map. XZ450 molecule is shown as sticks with carbon atoms in grey. Active site Mg^{2+} ions are shown as green spheres, water molecules as turquoise. The figure is presented in wall-eye stereo to aid viewing.

Supplementary Tables

Supplementary Table 1 | Structures of HIV-1 integrase strand transfer inhibitors (INSTIs), their related compound names ("compound") and identification numbers ("identification number") used in previous publications. (Color codes same as in Fig. 1: Hydrazines, Oxoisoindoles, Pyridinones, Diketo acids, Naphthyridines).

Compound	STRUCTURE	Identification number
**XZ15**	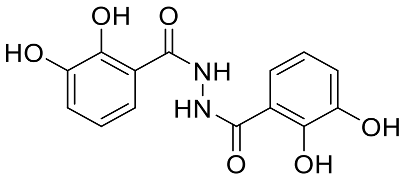 *N'*-(2,3-dihydroxybenzoyl)-2,3-dihydroxybenzohydrazide	**8b**[2]
**XZ45**	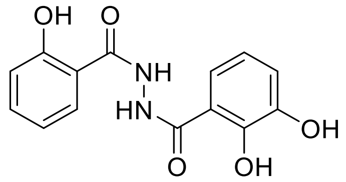 2,3-dihydroxy-*N'*-(2-hydroxybenzoyl)benzohydrazide	**8a**[2]
**XZ49**	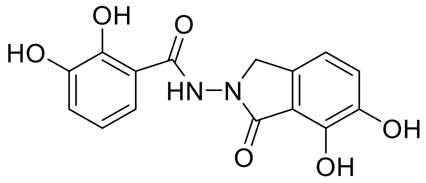 *N*-(6,7-dihydroxy-1-oxoisoindolin-2-yl)-2,3-dihydroxybenzamide	**22c**[2]
**XZ89**	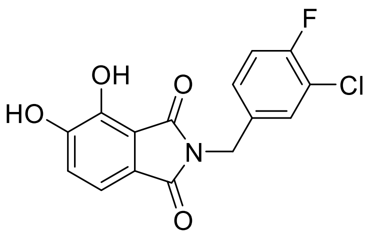 2-(3-chloro-4-fluorobenzyl)-4,5-dihydroxyisoindoline-1,3-dione	**17B**[3], **XZ89**[4]
**XZ90**	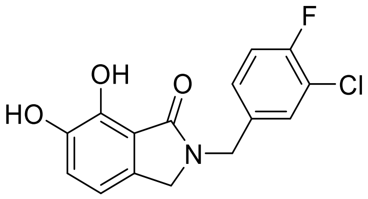 2-(3-chloro-4-fluorobenzyl)-6,7-dihydroxyisoindolin-1-one	**24e**[2], **17A**[3], **XZ90**[4]

**XZ202**	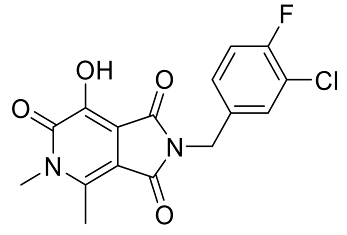<br>2-(3-chloro-4-fluorobenzyl)-7-hydroxy-4,5-dimethyl-1*H*-pyrrolo[3,4-*c*]pyridine-1,3,6(2*H*,5*H*)-trione	**5a**[5]
**XZ220**	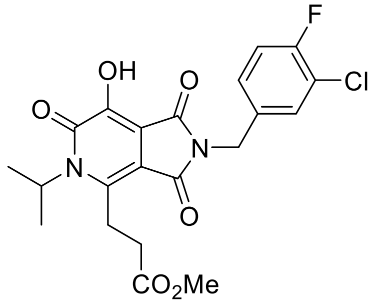<br>methyl 3-(2-(3-chloro-4-fluorobenzyl)-7-hydroxy-5-isopropyl-1,3,6-trioxo-2,3,5,6-tetrahydro-1*H*-pyrrolo[3,4-*c*]pyridin-4-yl)propanoate	**5e**[5]
**XZ235**	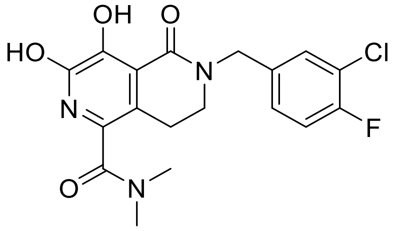<br>6-(3-chloro-4-fluorobenzyl)-3,4-dihydroxy-*N,N*-dimethyl-5-oxo-5,6,7,8-tetrahydro-2,6-naphthyridine-1-carboxamide	**XZ235**[6]
**XZ236**	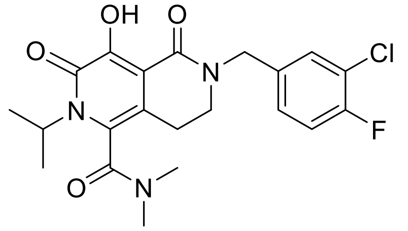<br>6-(3-chloro-4-fluorobenzyl)-4-hydroxy-2-isopropyl-*N,N*-dimethyl-3,5-dioxo-2,3,5,6,7,8-hexahydro-2,6-naphthyridine-1-carboxamide	**MK-0536**[7]
**XZ242**[*i*]	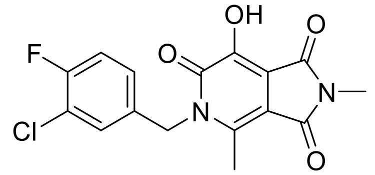<br>5-(3-chloro-4-fluorobenzyl)-7-hydroxy-2,4-dimethyl-1*H*-pyrrolo[3,4-*c*]pyridine-1,3,6(2*H*,5*H*)-trione	**XZ242**[5, 8]

**XZ256**[i]	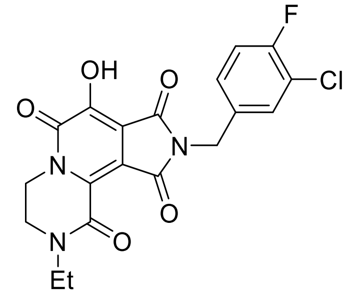<br>9-(3-chloro-4-fluorobenzyl)-2-ethyl-7-hydroxy-3,4-dihydropyrrolo[3',4':3,4]pyrido[1,2-*a*]pyrazine-1,6,8,10(2*H*,9*H*)-tetraone	**XZ256**[8, 9]
**XZ259**	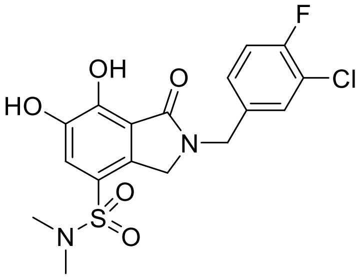<br>2-(3-chloro-4-fluorobenzyl)-6,7-dihydroxy-*N,N*-dimethyl-1-oxoisoindoline-4-sulfonamide	**XZ259**[4]
**XZ319**	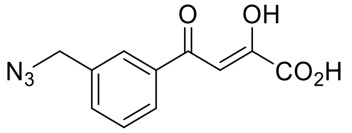<br>(*Z*)-4-(3-(azidomethyl)phenyl)-2-hydroxy-4-oxobut-2-enoic acid	**11**[10]
**XZ320**	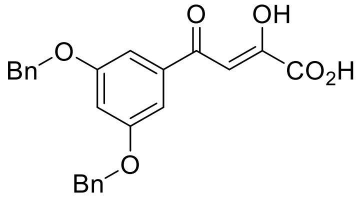<br>(*Z*)-4-(3,5-bis(benzyloxy)phenyl)-2-hydroxy-4-oxobut-2-enoic acid	**L-708, 906**[11]
**XZ351**	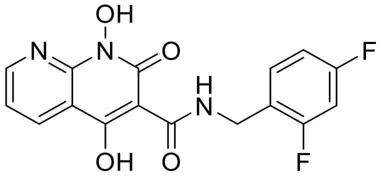<br>*N*-(2,4-difluorobenzyl)-1,4-dihydroxy-2-oxo-1,2-dihydro-1,8-naphthyridine-3-carboxamide	**8c**[12]
**XZ378**	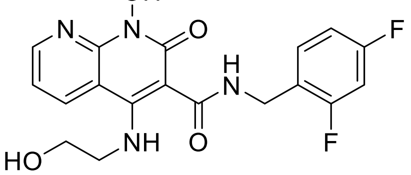<br>*N*-(2,4-difluorobenzyl)-1-hydroxy-4-((2-hydroxyethyl)amino)-2-oxo-1,2-dihydro-1,8-naphthyridine-3-carboxamide	**5o**[13]

**XZ384**	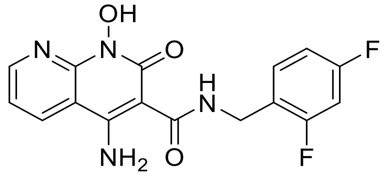<br>4-amino-*N*-(2,4-difluorobenzyl)-1-hydroxy-2-oxo-1,2-dihydro-1,8-naphthyridine-3-carboxamide	**5v**[13], **4a**[1]
**XZ413**	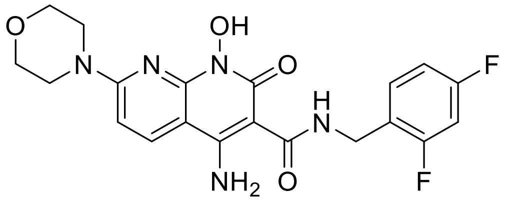<br>4-amino-*N*-(2,4-difluorobenzyl)-1-hydroxy-7-morpholino-2-oxo-1,2-dihydro-1,8-naphthyridine-3-carboxamide	**5f**[14]
**XZ419**	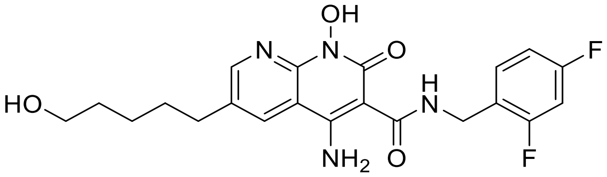<br>4-amino-*N*-(2,4-difluorobenzyl)-1-hydroxy-6-(5-hydroxypentyl)-2-oxo-1,2-dihydro-1,8-naphthyridine-3-carboxamide	**4c**[1]
**XZ420**[*i*]	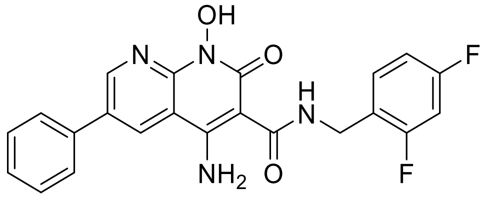<br>4-amino-*N*-(2,4-difluorobenzyl)-1-hydroxy-2-oxo-6-phenyl-1,2-dihydro-1,8-naphthyridine-3-carboxamide	**XZ420**[15]
**XZ423**	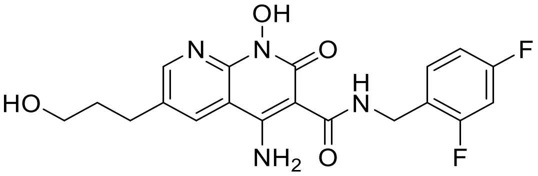<br>4-amino-*N*-(2,4-difluorobenzyl)-1-hydroxy-6-(3-hydroxypropyl)-2-oxo-1,2-dihydro-1,8-naphthyridine-3-carboxamide	**4b**[1]
**XZ426**	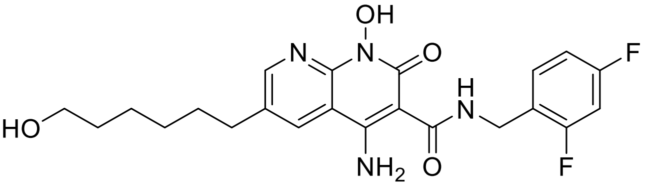<br>4-amino-*N*-(2,4-difluorobenzyl)-1-hydroxy-6-(6-hydroxyhexyl)-2-oxo-1,2-dihydro-1,8-naphthyridine-3-carboxamide	**4d**[1]

**XZ432**	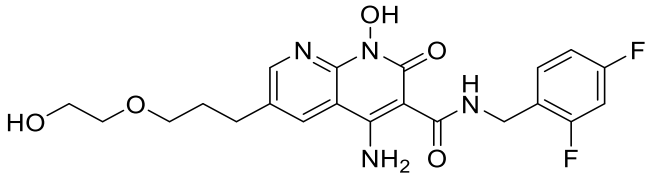<br>4-amino-*N*-(2,4-difluorobenzyl)-1-hydroxy-6-(3-(2-hydroxyethoxy)propyl)-2-oxo-1,2-dihydro-1,8-naphthyridine-3-carboxamide	**6h**[14]
**XZ434**	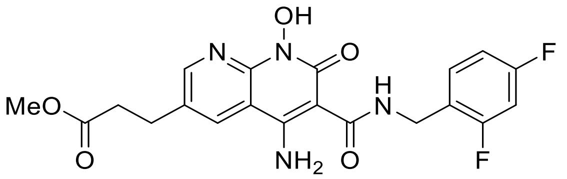<br>methyl 3-(5-amino-6-((2,4-difluorobenzyl)carbamoyl)-8-hydroxy-7-oxo-7,8-dihydro-1,8-naphthyridin-3-yl)propanoate	**6p**[14]
**XZ439**	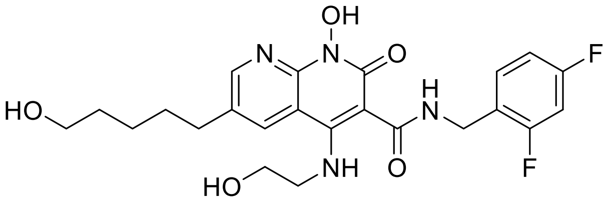<br>*N*-(2,4-difluorobenzyl)-1-hydroxy-4-((2-hydroxyethyl)amino)-6-(5-hydroxypentyl)-2-oxo-1,2-dihydro-1,8-naphthyridine-3-carboxamide	**6r**[14]
**XZ446**	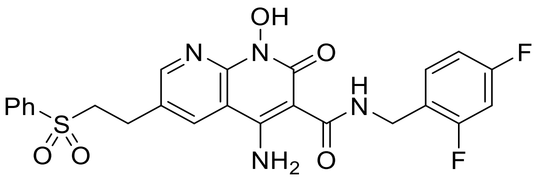<br>4-amino-*N*-(2,4-difluorobenzyl)-1-hydroxy-2-oxo-6-(2-(phenylsulfonyl)ethyl)-1,2-dihydro-1,8-naphthyridine-3-carboxamide	**4f**[1]
**XZ448**	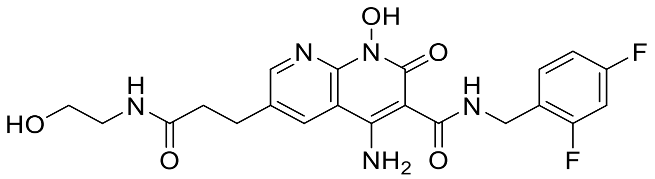<br>4-amino-*N*-(2,4-difluorobenzyl)-1-hydroxy-6-(3-((2-hydroxyethyl)amino)-3-oxopropyl)-2-oxo-1,2-dihydro-1,8-naphthyridine-3-carboxamide	**6n**[14]
**XZ450**	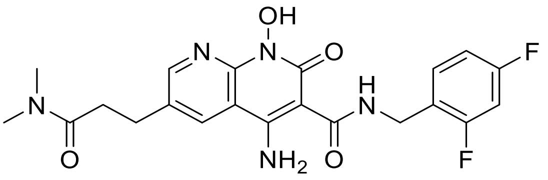<br>4-amino-*N*-(2,4-difluorobenzyl)-6-(3-(dimethylamino)-3-oxopropyl)-1-hydroxy-2-oxo-1,2-dihydro-1,8-naphthyridine-3-carboxamide	**6l**[14]

**XZ452**	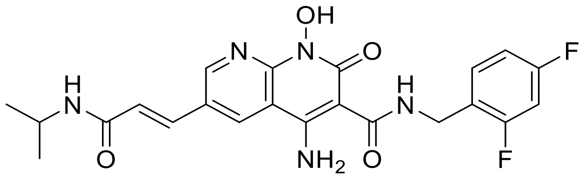 (*E*)-4-amino-*N*-(2,4-difluorobenzyl)-1-hydroxy-6-(3-(isopropylamino)-3-oxoprop-1-en-1-yl)-2-oxo-1,2-dihydro-1,8-naphthyridine-3-carboxamide	**6m**[14]
**XZ456**	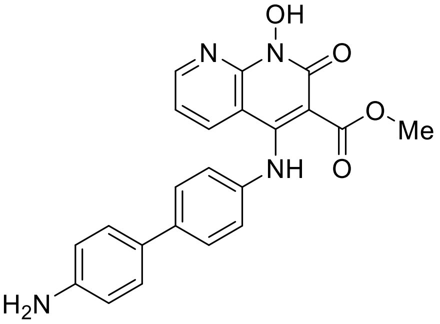 methyl 4-((4'-amino-[1,1'-biphenyl]-4-yl)amino)-1-hydroxy-2-oxo-1,2-dihydro-1,8-naphthyridine-3-carboxylate	**XZ456**[16]
**XZ460**	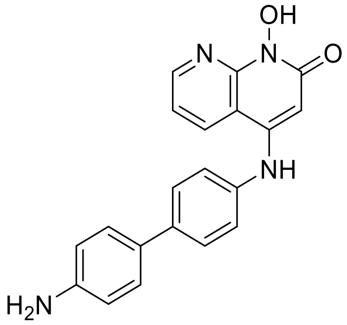 4-((4'-amino-[1,1'-biphenyl]-4-yl)amino)-1-hydroxy-1,8-naphthyridin-2(1*H*)-one	**XZ460**[16]
**XZ462**	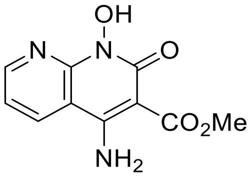 methyl 4-amino-1-hydroxy-2-oxo-1,2-dihydro-1,8-naphthyridine-3-carboxylate	**XZ462**[16]

Note: [i]Preparation details see Synthesis in Supplementary Materials and Methods.

Supplementary Table 2 | Summary of *in vitro* IC_{50} values determined with drug titrations in integrase strand-transfer assays alongside EC_{50} values for the best *in cellulo* performing INSTIs conducted in the cell-to-cell infection model. The drugs shown in green have been FDA-approved; other drugs are pre-approved INSTI candidates. The EC_{50} values for raltegravir and bictegravir are quoted as what we have recently reported in the same infection model. The cytotoxicity CC_{50} value obtained from existing literature is quoted in the right outermost column. Alongside, EC_{50} values obtained in an HIV-1 infection models are compared.

	HTLV-1		HIV-1	
	**IC_{50} ± stdev (nM)**	**EC_{50} ± stdev (nM)**	**EC_{50} ± stdev (nM)**	**CC_{50} (µM)**
**Raltegravir**	410 ± 78.5	6.42 ± 4.24[17]	9.4 ± 1.4[18]	>250[1]
**Bictegravir**	180 ± 29	0.3 ± 0.173[17]	1.6 ± 0.4[18]	>20[17]
**XZ423 (4b)**	155.3 ± 33.5	4.11 ± 1.52	3.1 ± 2.0[1]	>250[1]
**XZ450 (6l)**	120.2 ± 36.1	1.67 ± 1.52	5 ± 1.3[14]	>250[14]
**XZ446 (4f)**	115.4 ± 9.5	2.57 ± 0.81	2 ± 0.1[1]	>250[1]
**XZ448 (6n)**	131.9 ± 29.7	139 ± 81	263 ± 52[14]	>250[14]
**XZ452**	182.2 ± 44.7	N.D.		
**XZ426 (4d)**	162.8 ± 27.9	N.D.	2.3 ± 0.6[1]	>250[1]
**XZ434 (6p)**	203.7 ± 34.1	N.D.	0.67 ± 0.15[14]	>250[14]
**XZ432**	168 ± 17	N.D.		
**XZ384 (4a, 5v)**	210 ± 34	5.32 ± 2.36	1.1 ± 0.7[1]	>250[1,13]
**XZ419 (4c)**	225.7 ± 21	N.D.	1.3 ± 0.2[1]	>250[1]
**XZ420**	411 ± 93	N.D.		
**XZ439**	270 ± 82	N.D.		
**XZ378**	397 ± 64	N.D.		
**XZ236 (MK-0536; 2)**	322 ± 121	3.74 ± 1.42	17 ± 4[7]	>100[5]

Supplementary Table 3 | List of primers used to clone constructs for recombinant protein expression

Name	Sequence
GNM769	GGCCGAATTCCAGCTGAGTCCGGCAAAACTGCATAG
GNM770	GGCCCTCGAGTTAACCATGATGCTGATGATCACGTTC
GNM860	AAACAATCCGCAGCATCAGATGCCTCAAGGTCATATTCGTCGTGGTCTGCTGC
GNM861	GCAGCAGACCACGACGAATATGACCTTGAGGCATCTGATGCTGCGGATTGTTT
GNM862	CCTGGCAATTCGTCATACCACACATGTTCCGTATAATCCGACCAGCAGTGGTCT
GNM863	AGACCACTGCTGGTCGGATTATACGGAACATGTGTGGTATGACGAATTGCCAGG
GNM864	CGCGTCTGCCTCCGATTCCGGAAGAAAAACCGGTTACCACCAGTAAAACCCATT
GNM865	AATGGGTTTTACTGGTGGTAACCGGTTTTTCTTCCGGAATCGGAGGCAGACGC
GNM866	GCAGCTGCATCATAGTCCGCGTCTGGATCCGATTCCGGAAGCAAAACCGGTT
GNM867	AACCGGTTTTGCTTCCGGAATCGGATCCAGACGCGGACTATGATGCAGCTGC

Supplementary Table 4 | List of primers used for integrase strand-transfer and EMSA assays

Name	Sequence
STLV-1 MarB_U5_S30UP	TCTCTCCGGGAGAGAAGCGCCAAACACA
STLV-1 MarB_U5_S30UP	ACTGTGTTTGGCGCTTCTCTCCCGGAGAGA
HTLV-1 U5_S20Q_UP	GACTCACTATAGGGCACGCGTAGAGAAATTTAGTACACA
HTLV-1 U5_S20Q_B	ACTGTGTACTAAATTTCTCTACGCGTGCCCTATAGTGAGTC

Supplementary Table 5 | List of primers used for proviral load and Alu-qPCR determination.

Name	Sequence
tax forward	CGGATACCCAGTCTACGTGT
tax reverse	GAGCCGATAACGCGTCCATCG
gapdh forward	AACAGCGACACCCATCCTC
gapdh reverse	CATACCAGGAAATGAGCTTGACAA
Alu-F	CCTCCCAAAGTGCTGGGATTACA
Gag-R	GGCTTGGGTTTGGATGAGTA
Gag-F	CCCTCCAGTTACGATTTCCA

Supplementary Table 6 | Cryo-EM data collection and refinement.

	BIC	RAL	XZ450
**Database accession codes**			
EMDB	EMD-13077	EMD-13076	EMD-13075
RCSB	7OUH	7OUG	7OUF
**Data collection**			
Microscope	Titan Krios G3i	Titan Krios G3i	Titan Krios G3i
Operating voltage (kV)	300	300	300
Detector	Gatan K3	Gatan K3	Gatan K3
Physical pixel size (Å)	1.1	1.1	1.1
Defocus range (mm)	-0.7-3.6	-0.7-3.6	-0.7-3.6
Number of frames per movie	41	41	41
Total electron dose (e/Å^2)	50	50	50
Total movies acquired/used	8,502/6,129	17,744/14,271	9,188/7,719
Movie alignment software	MotionCor2	MotionCor2	MotionCor2
**Reconstruction**			
Software for 2D classification	cryoSPARC-2	cryoSPARC-2	cryoSPARC-2
Software for 3D classification	Relion-3.1	Relion-3.1	Relion-3.1
Software for reconstruction	Relion-3.1	Relion-3.1	Relion-3.1
Number of extracted particles	1,714,199	2,840,121	1,539,858
Number of refined particles	39,731	111,051	78,528
Symmetry imposed	C2	C2	C2
Map resolution (Å) [a]			
Overall	3.5	3.1	3.0
Active site region	2.8	3.0	2.5
Map 3DFSC sphericity	0.879	0.960	0.953
**Model refinement**			
Map sharpening B-factor (Å^2)	-79.3	-81.6	-75.7
Model composition			
Non-hydrogen atoms	15,088	15,090	15,090
Protein and DNA residues	1664/80	1664/80	1664/80
Metal ions (Zn, Mg)	4/4	4/4	4/4
Water molecules	6	8	12
INSTI	2	2	2
B-factors (Å^2)			
Protein	46.08	42.35	38.83
DNA	70.13	65.92	71.50
Ligands (INSTI)	22.84 (19.92)	13.36 (9.88)	11.33 (7.7)
Water	21.78	7.47	14.63
Software for real-space refinement	Phenix (dev_4155)	Phenix (dev_4155)	Phenix (dev_4155)
Real-space correlation coefficient	0.73	0.75	0.77
R.m.s. deviations			
Bonds (Å)	0.003	0.003	0.003
Angles (°)	0.538	0.703	0.547
Validation			
MolProbity score	1.40	1.32	1.34
Clash score	7.23	5.22	6.15
Rotamers outliers (%)	0	0.14	0.14
EMRinger score	2.68	2.84	2.55
Ramachandran plot quality (%)			
Favored	98.35	97.56	98.17
Disallowed	0	0	0

[a] Based on the FSC of 0.143 between half-sets

Supplementary References

1 Zhao, X. Z. *et al.* HIV-1 Integrase Strand Transfer Inhibitors with Reduced Susceptibility to Drug Resistant Mutant Integrases. *ACS Chem Biol* **11**, 1074-1081 (2016).

2 Zhao, X. Z. *et al.* 2,3-Dihydro-6,7-dihydroxy-1H-isoindol-1-one-Based HIV-1 Integrase Inhibitors. *J. Med. Chem.* **51**, 251-259, doi:10.1021/jm070715d (2008).

3 Zhao, X. Z. *et al.* Examination of halogen substituent effects on HIV-1 integrase inhibitors derived from 2,3-dihydro-6,7-dihydroxy-1H-isoindol-1-ones and 4,5-dihydroxy-1H-isoindole-1,3(2H)-diones. *Bioorg. Med. Chem. Lett.* **19**, 2714-2717, doi:10.1016/j.bmcl.2009.03.122 (2009).

4 Metifiot, M. *et al.* Activities, Crystal Structures, and Molecular Dynamics of Dihydro-1H-isoindole Derivatives, Inhibitors of HIV-1 Integrase. *ACS Chem. Biol.* **8**, 209-217, doi:10.1021/cb300471n (2013).

5 Zhao, X. Z. *et al.* Bicyclic hydroxy-1H-pyrrolopyridine-trione containing HIV-1 integrase inhibitors. *Chem. Biol. Drug Des.* **79**, 157-165, doi:10.1111/j.1747-0285.2011.01270.x (2012).

6 Han, W. *et al.* Preparation of hydroxynaphthyridinediones as HIV integrase inhibitors. WO2005087768A1 (2005).

7 Metifiot, M. *et al.* MK-0536 inhibits HIV-1 integrases resistant to raltegravir. *Antimicrob. Agents Chemother.* **55**, 5127-5133, doi:10.1128/AAC.05288-11 (2011).

8 Yan, Z. *et al.* HIV integrase inhibitors block replication of alpha-, beta-, and gammaherpesviruses. *mBio* **5**, e01318-01314 (2014).

9 Zhao, X. Z. *et al.* Development of tricyclic hydroxy-1H-pyrrolopyridine-trione containing HIV-1 integrase inhibitors. *Bioorg Med Chem Lett* **21**, 2986-2990 (2011).

10 Zhang, X. *et al.* Azido-Containing aryl β-Diketo acid HIV-1 integrase inhibitors. *Bioorg. Med. Chem. Lett.* **13**, 1215-1219, doi:10.1016/S0960-894X(03)00059-3 (2003).

11 Hazuda, D. J. *et al.* Inhibitors of strand transfer that prevent integration and inhibit HIV-1 replication in cells. *Science (Washington, D. C.)* **287**, 646-650, doi:10.1126/science.287.5453.646 (2000).

12 Zhao, X. Z. *et al.* Bicyclic 1-Hydroxy-2-oxo-1,2-dihydropyridine-3-carboxamide-Containing HIV-1 Integrase Inhibitors Having High Antiviral Potency against Cells Harboring Raltegravir-Resistant Integrase Mutants. *J. Med. Chem.* **57**, 1573-1582, doi:10.1021/jm401902n (2014).

13 Zhao, X. Z. *et al.* 4-Amino-1-hydroxy-2-oxo-1,8-naphthyridine-Containing Compounds Having High Potency against Raltegravir-Resistant Integrase Mutants of HIV-1. *J. Med. Chem.* **57**, 5190-5202, doi:10.1021/jm5001908 (2014).

14 Zhao, X. Z. *et al.* Structure-Guided Optimization of HIV Integrase Strand Transfer Inhibitors. *J. Med. Chem.* **60**, 7315-7332, doi:10.1021/acs.jmedchem.7b00596 (2017).

15 Zhao, X. Z. *et al.* Preparation of quinolinecarboxamide and naphthyridinecarboxamide compounds for inhibiting drug-resistant strains of HIV-1 integrase. WO2014186398A1 (2014).

16 Boyer, P. L. *et al.* Developing and evaluating inhibitors against the RNase H active site of HIV-1 reverse transcriptase. *J. Virol.* **92**, e02203-02217/02201-e02203-02217/02226, doi:10.1128/jvi.02203-17 (2018).
17 Barski, M. S., Minnell, J. J. & Maertens, G. N. Inhibition of HTLV-1 Infection by HIV-1 First- and Second-Generation Integrase Strand Transfer Inhibitors. *Front Microbiol* **10**, 1877, doi:10.3389/fmicb.2019.01877 (2019).
18 Tsiang, M. *et al.* Antiviral Activity of Bictegravir (GS-9883), a Novel Potent HIV-1 Integrase Strand Transfer Inhibitor with an Improved Resistance Profile. *Antimicrob Agents Chemother* **60**, 7086-7097, doi:10.1128/AAC.01474-16 (2016).
