# Supplementary material for: Structural basis for the inhibition of HTLV-1 integration inferred from cryo-EM deltaretroviral intasome structures
Source: Nat Commun. 2021 Aug 17;12:4996. doi: 10.1038/s41467-021-25284-1 (PMC8370991; doi:10.1038/s41467-021-25284-1)
Supplement: Supplementary file 3 — Reporting Summary [file 41467_2021_25284_MOESM3_ESM.pdf]

Corresponding author(s): Goedele N. Maertens

Last updated by author(s): Jul 12, 2021

Reporting Summary

Nature Portfolio wishes to improve the reproducibility of the work that we publish. This form provides structure for consistency and transparency in reporting. For further information on Nature Portfolio policies, see our Editorial Policies and the Editorial Policy Checklist.

Statistics

For all statistical analyses, confirm that the following items are present in the figure legend, table legend, main text, or Methods section.

n/a	Confirmed	
☐	☒	The exact sample size (*n*) for each experimental group/condition, given as a discrete number and unit of measurement
☐	☒	A statement on whether measurements were taken from distinct samples or whether the same sample was measured repeatedly
☐	☒	The statistical test(s) used AND whether they are one- or two-sided<br>*Only common tests should be described solely by name; describe more complex techniques in the Methods section.*
☒	☐	A description of all covariates tested
☒	☐	A description of any assumptions or corrections, such as tests of normality and adjustment for multiple comparisons
☐	☒	A full description of the statistical parameters including central tendency (e.g. means) or other basic estimates (e.g. regression coefficient) AND variation (e.g. standard deviation) or associated estimates of uncertainty (e.g. confidence intervals)
☐	☒	For null hypothesis testing, the test statistic (e.g. *F*, *t*, *r*) with confidence intervals, effect sizes, degrees of freedom and *P* value noted<br>*Give P values as exact values whenever suitable.*
☒	☐	For Bayesian analysis, information on the choice of priors and Markov chain Monte Carlo settings
☒	☐	For hierarchical and complex designs, identification of the appropriate level for tests and full reporting of outcomes
☒	☐	Estimates of effect sizes (e.g. Cohen's *d*, Pearson's *r*), indicating how they were calculated

Our web collection on statistics for biologists contains articles on many of the points above.

Software and code

Policy information about availability of computer code

Data collection	Cryo-EM movies were recorded using EPU 1.9.0 (Thermo Fisher Scientific). Gel images of EMSAs were collected using the Azure c600 imager, Coomassie stained and ethidium bromide stained gels were acquired using the BioRad GelDox XR+.
Data analysis	Quantification of bands in EMSA gels was done using ImageJ software version 1.50i and of the Coomassie and ethidium bromide stained gels the built in BioRad sofware (ImageLab 4.1) was used to determine the density of the bands using the raw image. Graphpad Prism 7 and 9 were used to calculate means, standard deviations and assess statistical significance (student t-test).<br>The micrograph movie frames were aligned, binned to the physical pixel size and summed, applying dose weighting as implemented in MotionCor2. Contrast transfer function (CTF) parameters were estimated using Gctf-v1.0645. At this stage, images with crystalline ice contamination were discarded; 14271 (intasome-RAL dataset), 6129 (BIC), and 7719 (XZ450) micrographs were retained for further processing. Particles were picked with Gautomatch-v0.53 (http://www.mrc-lmb.cam.ac.uk/kzhang/) using STLV intasome 2D class averages, low-pass filtered to 18A resolution, as templates. The particles, extracted in Relion-3.1 and binned to a pixel size of 4.4A, were subjected to two rounds of reference-free 2D classification in Cryo-SPARC-2. Particles belonging to well-defined 2D classes were re-extracted, binned 2-fold prior to 3D classification. This procedure yielded a single high-resolution 3D class per dataset; particles from the best 3D classes were re-extracted as full-sized images and used for 3D reconstruction using Relion-3.1 imposing C2 symmetry. Quality of the maps was further improved by CTF refinement (to estimate beam tilt and per-particles defocus) and Bayesian polishing, as implemented in Relion-3.1 Map resolutions were estimated using the gold-standard Fourier shell correlation (FSC) 0.143 criterion. Local resolutions of the cryo-EM maps were estimated with Blocres. To aid in model building process and to prepare figures, the maps were filtered and sharpened using deepEMhancer. For real-space refinements, the cryo-EM maps were sharpened and filtered using density modification procedure in Phenix using default parameters.<br><br>Intasome: INSTI model building and refinement<br>density modification was performed under default parameters in Phenix 1.18-384551, using half-maps and macromolecular sequence as inputs. Building was initiated with the model of the STLV-1 intasome:B56 complex, reduced to an asymmetric component corresponding to half-intasome. The IN A219E mutation was introduced in the model and the apparent conformational changes of some of the residues and

vDNA bases were modeled for in real-space in Coot 0.9.4. RAL and BIC structures were obtained from existing structures with accession codes 3OYA and 6RWM. The ligands were rigid-body fitted and locally refined. Density for the magnesium atoms was apparent and allowed their unambiguous positioning and water molecules were added to complete the Mg2+ coordination sphere. The model was duplicated and rigid-body docked in Chimera 1.12.0. to form the missing symmetrical part. The model was manually adjusted, and the final real-space refinement was conducted using Phenix version dev-4142 and the density modified map. Secondary structure restraints and base-pair/base stacking definitions based on the model, metal bond, ligand restraints and NCS constraints for the two halves of the symmetric nucleoprotein assembly were used. Quality of the final atomistic model was assessed with MolProbity and EMRinger. Pymol v1.8.0.3 was used to make the figures.

For manuscripts utilizing custom algorithms or software that are central to the research but not yet described in published literature, software must be made available to editors and reviewers. We strongly encourage code deposition in a community repository (e.g. GitHub). See the Nature Portfolio guidelines for submitting code & software for further information.

Data

Policy information about availability of data

All manuscripts must include a data availability statement. This statement should provide the following information, where applicable:

- Accession codes, unique identifiers, or web links for publicly available datasets
- A description of any restrictions on data availability
- For clinical datasets or third party data, please ensure that the statement adheres to our policy

The cryo-EM structures have been deposited with the Protein Data Bank and EMDB and are available under the following identifiers: STLV-1 intasome : XZ450 structure: 7OUF [https://www.rcsb.org/structure/7OUF] and EMD-13075 [https://www.ebi.ac.uk/pdbe/entry/emdb/EMD-13075]; STLV-1 intasome : RAL structure: 7OUG [https://www.rcsb.org/structure/7OUG] and EMD-13076 [https://www.ebi.ac.uk/pdbe/entry/emdb/EMD-13076]; and STLV-1 intasome : BIC structure: 7OUH [https://www.rcsb.org/structure/7OUH] and EMD-13077 [https://www.ebi.ac.uk/pdbe/entry/emdb/EMD-13077]. The authors declare that all other data supporting the findings of this study are available within the paper, its supplementary information files, and the Source Data provided with this paper.

Field-specific reporting

Please select the one below that is the best fit for your research. If you are not sure, read the appropriate sections before making your selection.

☒ Life sciences ☐ Behavioural & social sciences ☐ Ecological, evolutionary & environmental sciences

For a reference copy of the document with all sections, see nature.com/documents/nr-reporting-summary-flat.pdf

Life sciences study design

All studies must disclose on these points even when the disclosure is negative.

Sample size	No statistical methods were used to predetermine sample size. All biochemical assays were obtained from at least three independent biological replicates to ensure each data point was repeatable and comparable to other published studies. Sample size for the cryo-EM studies was determined by the availability of microscope time and to ensure unambiguous modeling of the structures. Sample sizes of the cryo-EM data sets are included in the Supplementary Table S6 and in the processing flow chart in Supplementary Figure S9.
Data exclusions	Cryo-EM data were processed in Relion-3.1 to exclude low-quality data (due to high drift or astigmatism)
Replication	Experiments were repeated at least three times and were successful. Raw data is proved in the Source Data accompanying the manuscript.
Randomization	Randomization is not applicable for macromolecular structure determination and was not used for the functional study since specific drugs were compared.
Blinding	Blinding is not relevant to this study, as no subjective allocation was involved in any of the structural and functional experiments.

Reporting for specific materials, systems and methods

We require information from authors about some types of materials, experimental systems and methods used in many studies. Here, indicate whether each material, system or method listed is relevant to your study. If you are not sure if a list item applies to your research, read the appropriate section before selecting a response.

Materials & experimental systems

n/a	Involved in the study	
☒	☐	Antibodies
☐	☒	Eukaryotic cell lines
☒	☐	Palaeontology and archaeology
☒	☐	Animals and other organisms
☒	☐	Human research participants
☒	☐	Clinical data
☒	☐	Dual use research of concern

Methods

n/a	Involved in the study	
☒	☐	ChIP-seq
☒	☐	Flow cytometry
☒	☐	MRI-based neuroimaging

Eukaryotic cell lines

Policy information about cell lines

Cell line source(s)	Jurkat E6.1 and MT-2 cells both originally obtained from ATCC
Authentication	None of the cell lines have been authenticated
Mycoplasma contamination	The cell lines were negative for mycoplasma contamination.
Commonly misidentified lines (See ICLAC register)	No commonly misidentified cell lines were used.
